# Long-Range Fit: A Software Package for the Representation and Study of Long-Range Molecular Interactions

**DOI:** 10.1021/acs.jctc.5c01984

**Published:** 2026-01-27

**Authors:** Adrian L. Batista-Planas, Ernesto Quintas-Sánchez, Richard Dawes

**Affiliations:** Department of Chemistry, 14717 Missouri University of Science and Technology, Rolla, Missouri 65409, United States

## Abstract

Describing intermolecular forces is fundamental to modeling and predicting the behavior of molecular systems. In particular, long-range molecular interactions—with electrostatic, induction, and dispersion as the main components—play a critical role, especially for low-temperature and low-density regimes. Long-range interactions are often described through perturbation theory, representing the electronic charge distribution via a multipolar series of the moments and polarizability tensors corresponding to each molecule. However, while the theory is well established, obtaining the resulting analytical expressions (and their practical implementation) constitutes a highly complex and system-dependent task. To address this challenge, we developed long-range-fit (LRF), an interactive and user-friendly software package designed to automate the generation and fitting of long-range interaction terms for arbitrary molecules in nondegenerate (ground or excited) electronic states. We have derived and implemented all terms up to 15th order, without approximations, via a spherical tensor representation, with symmetry adaptation to all molecular point-group symmetries. The resulting potential energy surface is compatible with most representations of the close interaction region.

## Graphical abstract

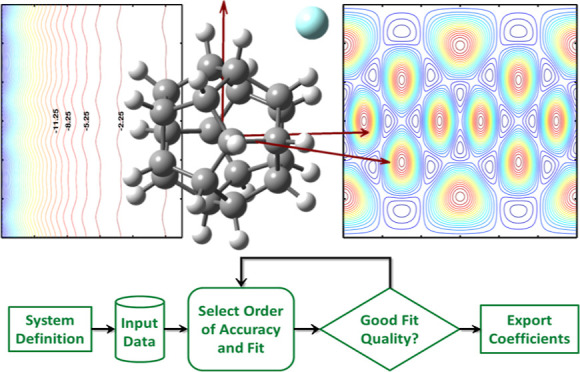

## 1 Introduction

The description of molecular interactions plays a crucial role in numerous scientific disciplines, with applications that span atmospheric chemistry, environmental chemistry, astrochemistry, and beyond.^1–6^ In particular, the importance of cold collisions—which take place in environments where the temperature ranges from perhaps a few to over a hundred Kelvin—has received increasing attention in the past decade, mainly due to significant theoretical and experimental advances in the field.^7–10^ Due to the rapid development of novel experimental techniques and theoretical approaches, the preparation/manipulation/simulation of a variety of species under cold conditions is a reality with growing capabilities, opening the door to scrutinize the complex chemistry taking place in remote parts of the Universe—such as the interstellar medium (ISM), circumstellar media, planetary atmospheres, and cometary comae.^11–13^ These efforts complement and guide the interpretation of the rapidly growing amount of data recorded by earth- and space-based telescopes.

For theoretical studies, the representation of a potential energy surface (PES) mapping the interaction energy with the corresponding configuration of the system, whether for a near-global space or a reduced region of interest—such as the region about the global minimum, along a reaction coordinate, or for some discrete set of geometries—often constitutes a crucial first step.^14–16^ Being a function of the coordinates of all atoms, the potential energy for a combined system of molecules can be separated into the intramolecular (the sum of the potential energies for each separated molecule) and intermolecular parts (the difference between the total and intramolecular potential energies). If the intermolecular PES is constructed using the rigid-rotor approximation, which usually works very well to describe nonreactive collisions of small molecules in low-temperature environments, the intramolecular energy becomes a constant, that of the separate monomers, and the dimensionality of the problem is significantly reduced: resulting in PESs being at most 6-dimensional for dimers, 12-dimensional for trimers, and so on. While 3- and higher-body interactions are of growing interest,^17,18^ most studies of the gas phase are limited to treating dimers, as is reasonable in low-pressure environments.

Although nowadays the energy for arbitrary points on the PES can usually be accurately obtained using high-level ab initio electronic structure methods—and used directly in molecular dynamics simulations, i.e., the “on-the-fly” approach—the computational cost makes this approach impractical in many cases.^19,20^ The common alternative is to compute a set of reference ab initio energies using a preferred/affordable level of theory, which provides the necessary accuracy, which is then used to fit an analytic PES. Methods to fit the data range from flexible or physically motivated single expansions to interpolative approaches, beginning with simple splines,^21^ as well as more sophisticated procedures such as interpolating moving least-squares^22–24^ and artificial intelligence/machine learning approaches.^16,25–28^ In the past decade, automated procedures that facilitate/minimize human intervention in the PES-construction process have also become popular, as implemented in programs such as AUTOSURF,^29^ AUTOPES,^30^ and ROBOSURFER.^31^ Our AUTOSURF package is typically employed to produce and refine a fit of the close interaction region, converging a specified configuration space to a specified error tolerance. However, depending on the problem of interest, not all regions of the PES play an equally important role. Given that statistically most gas-phase bimolecular collisions are not head-on, but rather have large impact parameters, then especially for interactions occurring at low temperatures with low collision energies, the system’s dynamics are largely determined by the long-range (LR) region of the PES.^32–34^ In the LR, small absolute errors can be large relative errors, with important implications.^35^ Thus, for theoretical studies of cold collisions, constructing a PES with the correct asymptotic behavior is an essential priority. This is the main focus of the present work: the representation and study of long-range molecular interactions.

There are three main contributions to the LR interactions, namely, electrostatic, induction (Debye interaction) and dispersion (London interaction). Numerous physics-based models have been developed in the last decades to represent LR interactions, such as the Morse-long-range method, a nonlinear fitting approach that combines radial potential functions with flexible parameters to enforce the well-known inverse power behavior into a single analytical function.^36–38^ Other approaches employ Morse-type variables to enforce slow variation in the LR, suppressing the oscillatory or divergent behavior of ordinary polynomials.^39^ Recently, machine learning approaches using different schemes and architectures such as feedforward^40,41^ and physics-informed neural networks^42,43^ have been introduced to the field. However, in terms of enforcing a physically correct behavior, methods based on the multipole expansion of the interaction potential are difficult to surpass.^44^

At sufficiently large separations, the interaction between two molecules (*A* and *B*) can be treated as two distinct charge distributions separated by the distance *R* between their respective centers-of-mass. In this scenario, the overlap between their wave functions can be ignored—since the error made by ignoring it decreases exponentially as *R* increases—and the system’s Hamiltonian Ĥ can be considered as a combination of ${Ĥ}^{0}$ , the Hamiltonian corresponding to the isolated molecules $\left({Ĥ}^{0}={Ĥ}^{A}+{Ĥ}^{B}\right)$ , and the operator ${Ĥ}^{\prime }$ , that describes the electrostatic interaction between the particles (nuclei and electrons) in different molecules. Since at large distances ${Ĥ}^{\prime }\ll {Ĥ}^{0}$ , it can be seen as a small perturbation in the system’s Hamiltonian. Then, since electron exchange can be neglected due to the molecular separation, the eigenfunctions of the unperturbed Hamiltonian ${Ĥ}^{0}$ can be written as simple products of the wave functions corresponding to the molecules *A* and *B* (Ψ_m_^A^Ψ_n_^B^) in a particular state *m* and *n*

1 $$\begin{array}{ll} {Ĥ}^{0}|mn\rangle & =\left({Ĥ}^{A}+{Ĥ}^{B}\right)|mn\rangle \\ & =\left(W_{m}^{A}+W_{n}^{B}\right)|mn\rangle \\ & =W_{mn}|mn\rangle , \end{array}$$

and standard Rayleigh–Schrödinger perturbation theory can be used to obtain the system’s total interaction energy for any particular nondegenerate state |pq⟩

2 $$W_{pq}=W_{pq}^{\left(0\right)}+W_{pq}^{\left(1\right)}+W_{pq}^{\left(2\right)}+...$$

where the (zeroth-order) energy *W*_pq_^(0)^ = *W*_p_^A^ + *W*_q_^B^ corresponds to the unperturbed state in which molecule *A* is in the excited state *p* (with energy *W*_p_^A^) and molecule *B* in the excited state *q* (with energy *W*_q_^B^); the first-order energy correction

3 $$W_{pq}^{\left(1\right)}=E_{elec}^{\left(pq\right)}=\langle pq|{Ĥ}^{\prime }|pq\rangle$$

is the expectation value of operator ${Ĥ}^{\prime }$ for state |*pq*⟩, which corresponds to the electrostatic energy of the system (*E*_elec_); while the second-order energy correction

4 $$W_{pq}^{\left(2\right)}=-\sum_{m,n}\frac{\langle pq|{Ĥ}^{\prime }|mn\rangle \langle mn|{Ĥ}^{\prime }|pq\rangle }{W_{m}^{A}+W_{n}^{B}-W_{p}^{A}-W_{q}^{B}}$$

the polarization energy, splits into the induction (*E*_ind_) and dispersion (*E*_disp_) energies

5 $$W_{pq}^{\left(2\right)}=E_{ind}^{A\left(pq\right)}+E_{ind}^{B\left(pq\right)}+E_{disp}^{\left(pq\right)}$$

where

6 $$E_{ind}^{A\left(pq\right)}=-\sum_{m\neq p}\frac{\langle pq|{Ĥ}^{\prime }|mq\rangle \langle mq|{Ĥ}^{\prime }|pq\rangle }{W_{m}^{A}-W_{p}^{A}}$$

7 $$E_{ind}^{B\left(pq\right)}=-\sum_{n\neq q}\frac{\langle pq|{Ĥ}^{\prime }|pn\rangle \langle pn|{Ĥ}^{\prime }|pq\rangle }{W_{n}^{B}-W_{q}^{B}}$$

8 $$E_{disp}^{\left(pq\right)}=-\sum_{\begin{array}{l} m\neq p\\ n\neq q \end{array}}\frac{\langle pq|{Ĥ}^{\prime }|mn\rangle \langle mn|{Ĥ}^{\prime }|pq\rangle }{W_{m}^{A}+W_{n}^{B}-W_{p}^{A}-W_{q}^{B}}$$

Notice that the sums in eqs 4 and 6–8 go over all possible states of the system, except for |*pq*⟩.

Without losing generality, we can describe the electrostatic interaction between the molecules ${Ĥ}^{\prime }$ considering the charge distribution *B* under the influence of an external nonuniform electric field $V^{A}\left(\mathrm{r⃗}\right)$ generated by molecule *A*: ${Ĥ}^{\prime }=\sum q_{b}V^{A}\left(\mathrm{b⃗}\right)$ ; where the sum goes for all particles *b* ∈ *B*, *q*_b_ represents the charge of particle *b* in position *b⃗* (with respect to the center-of-mass of molecule B). Assuming that $V^{A}\left(\mathrm{r⃗}\right)$ does not vary significantly over the whole charge distribution of molecule B, we can expand $V^{A}\left(\mathrm{b⃗}\right)$ , the potential in particle *b*, as a Taylor series around the center-of-mass of the molecule

9 $$V^{A}\left(\mathrm{b⃗}\right)=V^{A}\left(\mathrm{R⃗}\right)+\sum_{\alpha }V_{\alpha }^{A}\left(\mathrm{R⃗}\right)b_{\alpha }+\frac{1}{2}\sum_{\alpha ,\beta }V_{\alpha \beta }^{A}\left(\mathrm{R⃗}\right)b_{\alpha }b_{\beta }+...$$

where Greek letters (α and β) stand for the Cartesian coordinates (*x*, *y*, or *z*); *b*_α,β_ are the Cartesian coordinates of particle *b*; R⃗ is the vector from the center-of-mass of molecule *A* to the center-of-mass of molecule *B*; and we use the notation *V*_α_^A^ for ∂*V*/∂*b*_α_, *V*_αβ_^A^ for ∂^2^ *V*/∂*b*_α_∂*b*_β_, and so on. Then, we can write ${Ĥ}^{\prime }$ in terms of the multipole moments of molecule *B* interacting with the weakly varying electrostatic potential generated by molecule *A* as

10 $${Ĥ}^{\prime }=q^{B}V^{A}\left(\mathrm{R⃗}\right)+{\mathrm{\mu ̂}}_{\alpha }^{B}V_{\alpha }^{A}\left(\mathrm{R⃗}\right)+\frac{1}{3}{\mathrm{\Theta ̂}}_{\alpha \beta }^{B}V_{\alpha \beta }^{A}\left(\mathrm{R⃗}\right)+...$$

where we are using the Einstein summation convention: a repeated suffix implies summation over the three values (*x*, *y*, and *z*) corresponding to that suffix. In the first term, the monopole (zeroth-order) moment *q* ^B^ = ∑*q*_b_ is the total charge of molecule B; in the second term, the first-order moment μ_α_^B^ = ∑*q*_b_ *b*_α_ is the dipole moment; in the third term, the second-order moment $\Theta_{\alpha \beta }^{B}=\frac{1}{2}\sum q_{b}\left(3b_{\alpha }b_{\beta }-b^{2}\delta_{\alpha \beta }\right)$ is the quadrupole moment (δ_αβ_ is the Kronecker delta); and so on. Generalizing this notation, the 2^p^-pole moment operator for molecule B can be written as ${\mathrm{\xi ̂}}_{\alpha \beta ...\eta }^{B\left(p\right)}$ , a tensor of rank *p*, with *p* suffixes

11 $${\mathrm{\xi ̂}}_{\alpha \beta ...\eta }^{B\left(p\right)}=\frac{{\left(-1\right)}^{p}}{p!}\sum_{b}q_{b}b^{2p+1}\frac{\partial }{\partial b_{\eta }}\cdot \cdot \cdot \frac{\partial }{\partial b_{\beta }}\frac{\partial }{\partial b_{\alpha }}\left(\frac{1}{b}\right)$$

The same way, the 2^p^-pole moment operator for molecule A can be written as

12 $${\mathrm{\xi ̂}}_{\alpha \beta ...\eta }^{A\left(p\right)}=\frac{{\left(-1\right)}^{p}}{p!}\sum_{a}q_{a}a^{2p+1}\frac{\partial }{\partial a_{\eta }}\cdot \cdot \cdot \frac{\partial }{\partial a_{\beta }}\frac{\partial }{\partial a_{\alpha }}\left(\frac{1}{a}\right)$$

where the sum goes for all particles *a* ∈ *A*; and particle *a* is at position *a⃗* (with respect to the center-or-mass of molecule A) and has a charge *q*_a_. By using this notation, we can rewrite eq 10 in a more compact form

13 $${Ĥ}^{\prime }=\sum_{l=0}^{\infty }\frac{1}{\left(2l-1\right)!!}{\mathrm{\xi ̂}}_{\alpha \beta ...\eta }^{B\left(l\right)}V_{\alpha \beta ...\eta }^{A}\left(\mathrm{R⃗}\right)$$

where *n*!! = 1 × 3 × 5 ×...*n*. If we now expand $V^{A}\left(\mathrm{R⃗}\right)=\sum_{a}q_{a}/\left(4\pi \epsilon_{0}\left|\mathrm{R⃗}-\mathrm{a⃗}\right|\right)$ as a Taylor series about the center-of-mass of molecule *A*

14 $$V^{A}\left(\mathrm{R⃗}\right)=\sum_{k=0}^{\infty }\frac{{\left(-1\right)}^{k}}{\left(2k-1\right)!!}{\mathrm{\xi ̂}}_{\alpha \beta ...\eta }^{A\left(k\right)}T_{\alpha \beta ...\eta }^{\left(k\right)}$$

where the *T*-tensors $T_{\alpha \beta ...\eta }^{\left(n\right)}=\frac{1}{4\pi \epsilon_{0}}\nabla_{\alpha }\nabla_{\beta }...\nabla_{\eta }\left(1/R\right)$ ; we can finally obtain the electrostatic interaction ${Ĥ}^{\prime }$ in terms of the multipole moments of molecules A and B, the so-called multipole (or multipolar) expansion in Cartesian form^45^

15 $${Ĥ}^{\prime }=\sum_{l,k=0}^{\infty }C_{lk}{\mathrm{\xi ̂}}_{\alpha \beta ...\eta }^{A\left(k\right)}{\mathrm{\xi ̂}}_{\alpha^{\prime }\beta^{\prime }...\eta^{\prime }}^{B\left(l\right)}T_{\alpha \beta ...\eta \alpha^{\prime }\beta^{\prime }...\eta^{\prime }}^{\left(k+l\right)}$$

where *C*_lk_ = (−1)^*k*^/[(2*k* – 1)!!(2*l* – 1)!!].

The different energy contributions to the LR interactions can be calculated by substituting the multipole expansion in eqs 3 and 6–8. This way, the electrostatic energy for state |*pq*⟩ will be the expectation value of ${Ĥ}^{\prime }$ (cf. eq 3) and can be obtained by simply replacing each multipole operator in eq 15 by its expectation value for the state of interest

16 $$E_{elec}=\sum_{l,k=0}^{\infty }C_{lk}\xi_{\alpha \beta ...\eta }^{A\left(k\right)}\xi_{\alpha^{\prime }\beta^{\prime }...\eta^{\prime }}^{B\left(l\right)}T_{\alpha \beta ...\eta \alpha^{\prime }\beta^{\prime }...\eta^{\prime }}^{\left(k+l\right)}$$

For simplicity, and to avoid overloading the notation, here and in the following, we do not include another set of indices (*p*, *q*) specifying the particular state of the system—e.g., when referring to the expectation value of the multipole operators: ξ = ⟨pq|ξ̂|pq⟩. Notice how each term in the sum depends on a specific combination of the multipole moments corresponding to the charge distributions A and B. It is also worth mentioning that although both ξ- and *T*-tensors are related to the *n*th-gradient of a Cartesian vector, they have a critical difference: ξ_αβ...η_^(*n*)^ are constant, related to the *n*th-gradient of a “local” particle position, describing a fixed picture of the charge distribution of each molecule with respect to its center-of-mass; the *T*-tensors, on the other hand, have a functional dependence on the separation and relative orientation of the two molecules.

Since the *T*-tensors are computed as successive derivatives of 1/*R*, *T*_αβ...η_^(*n*)^ will be proportional to *R* ^–(*n*+1)^, and by regrouping the terms by the same power of 1/*R*, eq 16 can be conveniently written as

17 $$\begin{array}{ll} E_{elec}=T^{\left(0\right)}q^{A}q^{B}+ & \propto R^{-1}\\ T_{\alpha }^{\left(1\right)}\left(q^{A}\mu_{\alpha }^{B}-q^{B}\mu_{\alpha }^{A}\right)+ & \propto R^{-2}\\ T_{\alpha \beta }^{\left(2\right)}\left(\frac{1}{3}q^{A}\Theta_{\alpha \beta }^{B}-\mu_{\alpha }^{A}\mu_{\beta }^{B}+\frac{1}{3}\Theta_{\alpha \beta }^{A}q^{B}\right)+ & \propto R^{-3}\\ T_{\alpha \beta \gamma }^{\left(3\right)}\left(\frac{1}{15}q^{A}\Omega_{\alpha \beta \gamma }^{B}-\frac{1}{3}\mu_{\alpha }^{A}\Theta_{\beta \gamma }^{B}+\frac{1}{3}\Theta_{\alpha \beta }^{A}\mu_{\gamma }^{B}-\frac{1}{15}\Omega_{\alpha \beta \gamma }^{A}q^{B}\right) & \propto R^{-4}\\ +... \end{array}$$

where the various multipole–multipole interactions are grouped according to their *R*-dependence, with the *n*th term in the expansion (or *n*th “order”) being proportional to *R* ^–*n*^. Similar expressions can be obtained for the dispersion and induction energies
18

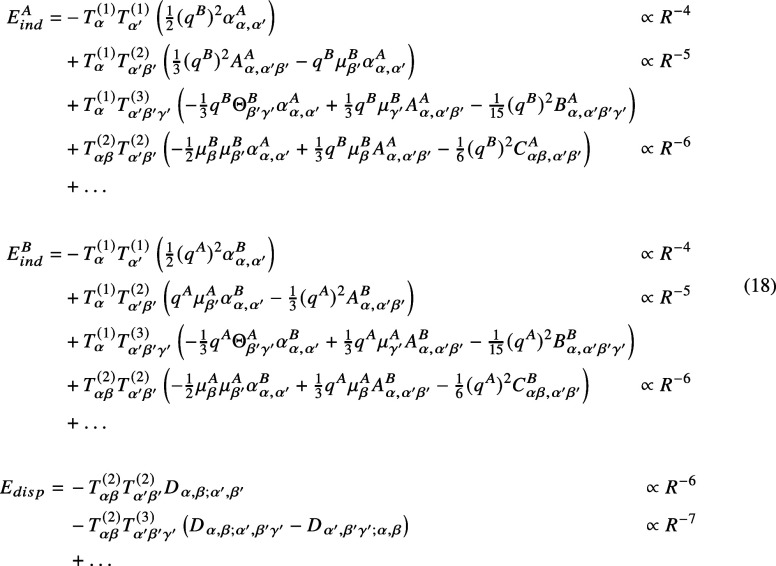

where α represents the dipole–dipole polarizabilities, *A* is the dipole–quadrupole polarizabilities, *B* is the dipole–octupole polarizabilities, *C* is the quadrupole–quadrupole polarizabilities, and *D* is the dispersion coefficients. This way, the terms in the multipolar expansion are arranged according to their potential importance at various distances with each contribution clearly identified/separated.

At very large *R*, the lowest-rank moments and polarizabilities are most important, but the others may become increasingly significant as *R* decreases, depending on their coefficients. For example, at large distances, the most relevant term for the electrostatic interaction between two ions is proportional to 1/*R* (the charge–charge interaction), but for neutral polar molecules, the leading (first nonzero) term corresponds to the dipole–dipole interaction (∝1/*R* ^3^), while for two nonpolar molecules, the leading interaction might be the quadrupole–quadrupole term, proportional to 1/*R* ^5^. For higher symmetry cases, the leading term can be an even higher moment, such as for dimers of tetrahedral molecules (e.g., methane), where the leading term corresponds to the octupole–octupole interaction (∝1/*R* ^7^), or for two fullerene molecules (*I*_h_ symmetry), where the leading electrostatic term is given by the interaction between the 2^6^-pole of each molecule (∝1/*R* ^13^). Systems with higher-order leading terms are relatively common, including H_2_ and ethylene, which both have vanishing dipole and octupole moments, making the leading moments quadrupole and hexadecapole (2^4^-pole). Note that for neutral molecules, induction and dispersion interactions both start at sixth-order, and for ions, induction appears even sooner, at fourth-order, thus making these other types of interaction potentially as significant—or more so—than electrostatics. This can be the case when leading electrostatic terms are nonzero but small, such as for the CO-dimer, which will be discussed in more detail later, or for the example of two fullerene molecules just mentioned above.

Each term in the expansion for each interaction has a clear physical meaning. The magnitude of the terms in the electrostatic interactions depends on the values of multipole moments; the terms in the induction and dispersion expansions are proportional to the molecular polarizabilities. In most applications, the main challenge will be the precise evaluation of each of the interactions for a given order of the multipole expansion. This raises the issue of how to obtain values for the electrostatic moments and polarizability tensors to implement in the expressions. Experimentally determined data are only available for a limited number of systems and are usually confined to the leading electrostatic moment(s) and perhaps the dipole polarizability. Values can be obtained directly from electronic structure calculations but are not so easily extracted from the high levels of theory commonly employed in constructing PESs (e.g., CBS-extrapolated coupled cluster theory with perturbative triples or even higher-order correlation treatment). To do so, one would need to compute extrapolated energies within a series of finite-field calculations, formulas that become increasingly complicated and numerically sensitive for higher moments.^46,47^ As we will discuss later, our chosen strategy is simply to fit them using ab initio energies from the LR region of the PES.

The choice of representation has a large impact on the total number of coefficients and therefore the total number of terms to be handled in the multipole expansion. Due to its simplicity, the Cartesian representation described by eqs 17 and 18 is the most straightforward to develop/understand, and as a consequence, it is the most commonly employed. However, the Cartesian representation has a severe drawback. The number of components for each higher multipole moment increases rapidly with order, which soon becomes unmanageable. Cartesian tensors ξ^(p)^ have rank p, and consequently, each has 3^p^ components/coefficients. For the electrostatic energy, for example, this means (cf. eq 17) that the total number of new coefficients increases exponentially as ∼3^*n*–1^ with the *n*-th order in the multipole expansion. Although only 2*n* – 1 of them are linearly independent for each molecule (and therefore only 4*n* – 2 need to be computed or known a priori for each new order), the complete set of coefficients still needs to be implemented in the analytical formulas, making the total number of terms increase excessively. Thus, for any practical application, to include high-order terms of the multipole expansion employing a Cartesian representation quickly becomes an infeasible task: to include—for the electrostatic interactions alone—all terms up to sixth-order, a total of 728 coefficients need to be determined; at eighth-order, there are 6560 coefficients; at tenth-order, there are around 59 thousand coefficients; and at 15th-order, the total number of coefficients to be included in the multipole expansion would be close to 14.3 million. It is worth emphasizing that this is only considering the electrostatic interactions: at 15th-order, there are also ∼14 million coefficients in the induction expansion and another ∼300 million in the dispersion energy expansion that would need to be considered. Given that the issue comes from the components of ξ^(*p*)^ not being linearly independent, the solution is straightforward: switch from Cartesian to a representation in spherical coordinates—an irreducible representation, which for each moment has no linear dependencies, so the number of coefficients involved grows only linearly with the order—the trade-off: the mathematical complexity of those fewer terms is considerably higher. Using the same example as before, up to sixth-order (for the electrostatic interactions), there are 72 components in the spherical form of the multipole expansion, which is about a factor of 10 fewer than in Cartesian coordinates. Moreover, this reduction in the number of terms with respect to the Cartesian formulation is exponentially more significant as the order of the expansion increases—at the 15th-order, there are only 450 coefficients in the electrostatic expansion—making the spherical formulation, as can be seen in Figure 1, dramatically more efficient as higher orders are included.

**Figure 1:**
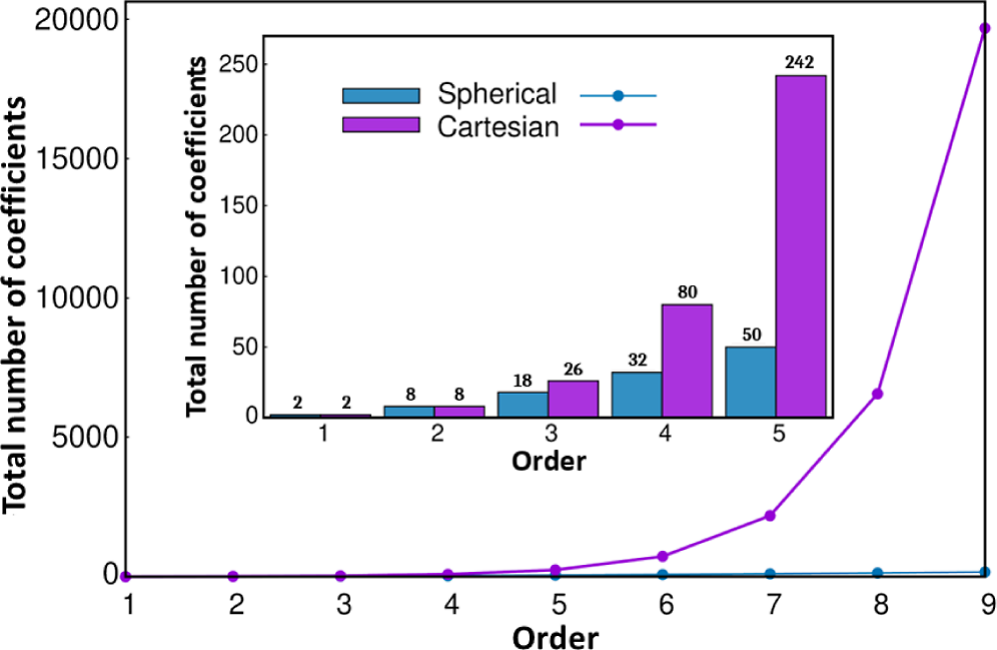
Comparison between the total number of electrostatic coefficients as a function of the order of the multipole expansion in the Cartesian and spherical representations.

The fundamental theory of intermolecular forces is well established. The mathematical framework for describing long-range interactions based on the multipole expansion has been known for decades, even for molecules in excited states, systems with degenerate electronic states, and higher-order corrections to the perturbation treatment such as the hyperpolarizability contribution.^45,48–50^ However, the general implementation for specific systems can be challenging, considering both the growing number of terms with the order in the multipole expansion and the complexity of the analytical expressions for each term. Additionally, the general formulas depend on the symmetry of the system—molecules with lower symmetry have more nonzero moments, many more coefficients and terms, and therefore a more complex multipole expansion. Further considerations should be made if the two molecules are identical or chiral partners. This is why in practice in the construction of global PESs, treatment of the LR interactions has often been an afterthought, where a switch to a simple LR model is added based on at most a few leading electrostatic terms, in a system-specific case-by-case basis.

The software package AUTOPES^30,51^ is one notable exception. Their approach is a symmetry adapted perturbation theory (SAPT)-based representation of both the short- and long-range interaction regions. Site–site interactions, termed a distributed expansion, are used to represent the close interaction region, and the number of sites in each fragment, including possible off-atom sites, is used to tune the accuracy of the fit. For the LR, they also employ the distributed expansion, but the fit is informed by consideration of the expected behavior of an asymptotic multipole-based expansion as described in their 2016 paper.^30^ In their approach, multipoles and polarizabilities are computed for each monomer, and then a constructed set of terms is used to generate energies, which are in turn used to fit the distributed expansion. For the long range, the accuracy of their approach depends on the fitting data (how accurately the moments and polarizabilities can be obtained via the SAPT method) as well as how accurately the distributed expansion can accommodate the topography computed by their multipole-based expansion.

In 2024, Yu et al. presented a multipole-based package for representation of the LR interaction between two molecules in their nondegenerate electronic ground states. Written in FORTRAN, the program ABLRI^52^ contains system-dependent subroutines that can be modified by the user to obtain the LR expansion with six possible types of symmetry for each fragment (*C*_1_, *C*_s_, *C*_2v_, *C*_∞v_, *D*_∞h_, and spherical), provided the required components of the electrostatic moments and polarizabilities are known/computed a priori and used as input. Using the Cartesian representation, electrostatic interactions are included up to the fifth-order and induction interactions up to the sixth-order (if both monomers have zero charge, then electrostatic interactions are included up to sixth-order and induction interactions up to eighth-order), while the dispersion energy is estimated (up to eighth-order) using the Unsöld approximation^53,54^ instead of the corresponding polarizability expansion. A recently developed approach for treating cases where one or both fragments are in a degenerate electronic state was reported by Yu and collaborators,^55^ based on degenerate perturbation theory. There, a multipole-based LR expansion was implemented to construct the potential energy matrix for two test cases, systems in their ground but degenerate electronic states: O­(^3^P)–OH­(^2^Π), with electrostatic interactions up to sixth-order, induction interactions up to seventh-order, and dispersion interactions up to seventh-order; and OH­(^2^Π)–OH­(^2^Π), with electrostatic interactions up to fifth-order, induction interactions up to seventh-order, and dispersion interactions up to seventh-order. Since only the first few terms were included for each interaction—and also due to the symmetry of the fragments—both the Cartesian and spherical representations were able to be satisfactorily implemented and compared. This is a promising step in an important area where couplings between the electronic state components can reflect complicated behaviors and topographies within the manifold of states. It will be interesting to see how this description of the long-range meshes with various diabatization protocols used to treat the close interaction region given that most diabatization schemes are somewhat ad hoc and damping is often applied.

Our first approach to this problem used the Cartesian framework, and it was a significant effort to include in the LR expansion—for electrostatic, induction, and dispersion interactions—all terms up to the sixth-order (and some seventh-order terms) for seven different symmetries: *C*_1_, *C*_s_, *C*_2v_, *C*_3v_, C_∞v_, *D*_∞h_, and spherical. Furthermore, that implementation was restricted to ground electronic states. Our goal was to go well beyond this and develop a general methodology that could be used to describe LR intermolecular interactions between two molecules with arbitrary symmetries, either in their ground or excited (nondegenerate) electronic states, with rigorous inclusion of high-order terms in the expansion. There were four main challenges that needed to be addressed: (1) given the electronic state, symmetry, and charge of each molecule, identify the relevant moments, polarizabilities, and dispersion coefficients and construct, for each interaction, the arrangement of terms to be included in the LR expansion—i.e., obtain the system-specific equivalent of eqs 17 and 18; (2) derive the analytical expression (depending on the intermolecular coordinates) for each of the terms in this system-specific expansion; (3) obtain all the coefficients included in the expansion; and (4) produce user-friendly subroutines that could be used to reproduce the constructed LR-PES in various sets of coordinates—ideally black-box, rapid to evaluate, and portable (written in FORTRAN or other popular language), so that it can easily be used by other codes, or combined with other representations of the close interactions to provide a global description of the PES. Here, we introduce our solution to these challenges, released as a freely available software package named long-range-fit (LRF). This first release is designed to treat systems composed of two arbitrary molecules in their ground or excited (nondegenerate) states.

Based on the spherical representation, LRF is designed to automatically generate the complete analytical LR expansion (up to 15th-order) for any system composed of two neutral or charged molecules—in a ground or excited (nondegenerate) state—and determine the coefficients by fitting to ab initio data. Whether they are different, identical, or chiral partners, the symmetry of the system is respected and enforced. To provide insight into the nature of the most important interactions, tables of all relevant terms (classified as electrostatic, induction, and dispersion) are produced based on the charge and point group symmetry for each of the molecules. For each fragment, a fully exhaustive list of all molecular point group symmetries is available, covering every possible scenario. If enough quantities (multipole, polarizability, and dispersion coefficients) are accurately known/computed for each fragment, then those can be directly inputted as fixed parameters and LRF can render a useable representation of the long-range interactions, truncated to any chosen order. More commonly, the molecular parameters are not known accurately, if at all. This is where LRF shines. Given a limited set of single-point energies distributed arbitrarily over the long-range region of the PES—often, but not necessarily, computed at the same level of theory as in the close interaction region—LRF will fit the data using all relevant terms up to a user-specified maximum order. No finite field calculations nor special grids are required. There are no missing terms up to the specified order; the expressions have correct symmetry constraints, and each term has its proper angular and distance dependencies. Beyond producing fits, LRF includes a suite of tools to analyze the quality of those fits and systematically investigate the role of different types of interaction and multipole orders in the long-range behavior of molecular systems. This way, LRF functions both as a practical fitting engine and as a platform for exploring the physics encoded in long-range interactions. LRF will also export a FORTRAN subroutine for external evaluation of the fitted potential, which can also be easily and smoothly connected to any preferred representation of the close interaction region, contributing to providing a global description of the PES.

The rest of the article is organized as follows. In Section 2, we describe the strategy and underlying algorithms implemented in LRF—only the most important aspects are highlighted here; additional details will be discussed in depth in a future paper. In Section 3, the program is introduced, and its main features and capabilities are demonstrated using illustrative examples. The final section provides a summary and concluding remarks.

## 2 Methodology

### 2.1 Coordinate System

The coordinate system used is shown in Figure 2. We start by selecting three different frames of reference: one molecule-fixed (MF) frame attached to each molecule, with its origin at its center-of-mass; and a dimer-fixed (DF) frame, with its origin at the center-of-mass of molecule A and its *z*-axis pointing at the center-of-mass of molecule B. The orientation of each MF frame can be chosen arbitrarily—the principal axes of the moment of inertia tensor of the molecule is a common choice. However, we will explain later how the orientation of the MF frame should be specified to enable use of the molecule’s relevant symmetry-elements in our program. Based on these definitions, two sets of Euler angles, Ω_A_ = (α_A_, β_A_, γ_A_) and Ω_B_ = (α_B_, β_B_, γ_B_), can be used to specify the orientation of each MF frame with respect to the DF frame, where α and β are the azimuth and polar angles of each molecule, respectively, and γ describes the rotation about their *z*-axis. When Ω_A_ = Ω_B_ = (0, 0, 0), all *z*-axes are aligned and all *x*- and *y*-axes are parallel. This way, the intermolecular coordinates can be chosen as (*R*, Ω_A_, Ω_B_), where *R* is the distance between the molecular centers-of-mass. Note that not all of the Euler angles are independent: since the first rotation of both molecules is around the *z*-axis of the DF frame, it suffices to know only the difference or phase α = α_A_ – α_B_ between the azimuthal angles. Furthermore, the number of angular coordinates is also reduced, depending on the structure of the molecules, as shown in Table 1.

**Figure 2:**
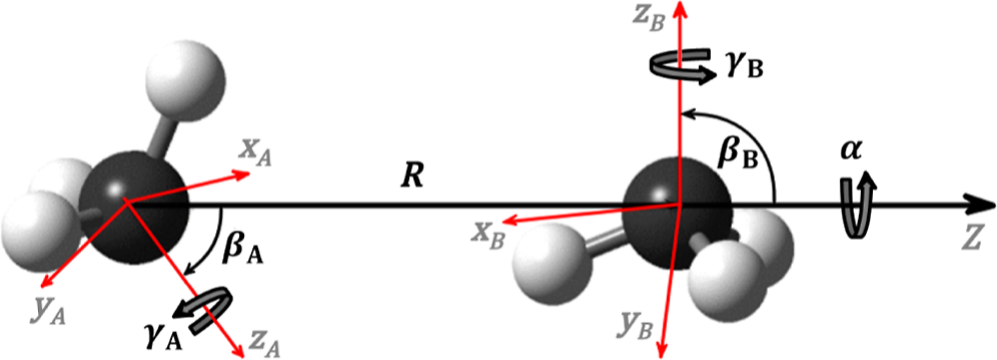
Intermolecular coordinates (*R*, α, β_A_, β_B_, γ_A_, γ_B_). For clarity, the *X-* and *Y*-axes of the DF frame have been omitted.

**Table 1:** Angular Coordinates Employed by LRF and Corresponding Dimensionality of the System

Dim.	molecule A	molecule B	angular coordinates
1D	atom	atom	-
2D	linear	atom	β_A_
3D	nonlinear	atom	β_A_, γ_A_
4D	linear	linear	α, β_A_, β_B_
5D	nonlinear	linear	α, β_A_, β_B_, γ_A_
6D	nonlinear	nonlinear	α, β_A_, β_B_, γ_A_, γ_B_

### 2.2 Spherical Tensor Formulation

In the spherical formulation, the electrostatic interaction operator ${Ĥ}^{\prime }$ in the DF (space-fixed) frame can be written as^45^

19 $${Ĥ}^{\prime }=\sum_{l_{a},l_{b}}\;\sum_{m_{a}^{\prime },m_{b}^{\prime }}{\mathrm{Q̂}}_{l_{a},m_{a}^{\prime }}^{A\left(DF\right)}{\mathrm{Q̂}}_{l_{b},m_{b}^{\prime }}^{B\left(DF\right)}X_{m_{a}^{\prime },m_{b}^{\prime }}^{l_{a},l_{b}}\left(\mathrm{R⃗}\right)$$

where ${\mathrm{Q̂}}_{l,m^{\prime }}^{\left(DF\right)}$ represent the components of the 2^*l*^-pole moment operators for each molecule, which depend on the polar and azimuthal angular coordinates (θ_a,b_, φ_a,b_) of each particle *a* ∈ *A* and *b* ∈ *B*

20 $$\begin{array}{ll} {\mathrm{Q̂}}_{l_{a},m_{a}^{\prime }}^{A\left(DF\right)} & =\sum_{a}q_{a}a^{l_{a}}\sqrt{\frac{4\pi }{2l_{a}+1}}Y_{l_{a},m_{a}^{\prime }}\left(\theta_{a},\phi_{a}\right)\\ {\mathrm{Q̂}}_{l_{b},m_{b}^{\prime }}^{B\left(DF\right)} & =\sum_{b}q_{b}b^{l_{b}}\sqrt{\frac{4\pi }{2l_{b}+1}}Y_{l_{b},m_{b}^{\prime }}\left(\theta_{b},\phi_{b}\right), \end{array}$$

and

21 Xla,lbma′,mb′(R⃗)=(la×lb×la+lbma′×mb′×ma′+mb′)(2la+2lb+1)!(2la)!(2lb)!×14πϵ0(−1)laIla+lb,ma′+mb′(R⃗),

where the expression in large brackets is a Wigner 3-*j* symbol and $I_{l,m}\left(\mathrm{R⃗}\right)=R^{-l-1}\sqrt{4\pi /\left(2l+1\right)}Y_{lm}\left(\theta ,\varphi \right)$ are irregular spherical harmonics. The multipole moment operators in the DF frame are related to those in the MF frame

22 $${\mathrm{Q̂}}_{lm^{\prime }}^{A,B\left(DF\right)}=\sum_{m}{\mathrm{Q̂}}_{lm}^{A,B}D_{mm^{\prime }}^{l}\left(\Omega_{A,B}^{-1}\right)$$

where Ω = (α, β, γ) is the rotation that takes the global DF axes to the local MF axes, and $D_{mm^{\prime }}^{l}$ are the Wigner rotation matrix elements. Since we will be only using local axes to describe the multipole operators from now on, we do not include another superscript (MF) to avoid overloading the notation.

Now, defining analogues to the *T*-tensors of the Cartesian formulation

23 $$T_{m_{a},m_{b}}^{l_{a},l_{b}}=\sum_{m_{a}^{\prime }m_{b}^{\prime }}X_{l_{a},l_{b}}^{m_{a}^{\prime },m_{b}^{\prime }}\left(\mathrm{R⃗}\right)D_{m_{a}m_{a}^{\prime }}^{l_{a}}\left(\Omega_{A}^{-1}\right)D_{m_{b}m_{b}^{\prime }}^{l_{b}}\left(\Omega_{B}^{-1}\right)$$

we can rewrite eq 19 in a more convenient notation

24 $${Ĥ}^{\prime }=\sum_{\begin{array}{l} l_{a},l_{b}\\ m_{a},m_{b} \end{array}}{\mathrm{Q̂}}_{l_{a},m_{a}}^{A}{\mathrm{Q̂}}_{l_{b},m_{b}}^{B}T_{m_{a},m_{b}}^{l_{a},l_{b}}\left(R,\Omega_{A},\Omega_{B}\right),$$

where the multipole moment operators are now referred to local molecular axes (different for each molecule) and the angular and distance dependence is all contained in the *T*-tensors. This can be abbreviated even further if we rearrange the series following the notation introduced by Stone^45^

25 $${Ĥ}^{\prime }=\sum_{t}\;\sum_{u}{\mathrm{Q̂}}_{t}^{A}{\mathrm{Q̂}}_{u}^{B}T_{t,u}\left(R,\Omega_{A},\Omega_{B}\right)$$

in which t and u refer to the members of the series of angular momentum labels: {00, 10, 11*c*, 11*s*, 20, 21*c*, 21*s*, 22*c*, 22*s*, 30, ...}. These labels correspond to *lm*, with *c* and *s* (cosine and sine) designating the positive and negative projections of *m,* respectively. This way, for example, the *T*_20,11*c*_ component of the *T*-tensor represents the interaction between the component *m*_a_ = 0 of the spherical quadrupole (*l*_a_ = 2) of molecule *A* and the component *m*_b_ = 1*c* of the dipole moment (*l*_b_ = 1) of molecule *B*. By substituting this multipole expansion of ${Ĥ}^{\prime }$ in eq 3, and eqs 6–8, the different energy contributions to the LR interactions can be obtained in the spherical tensor formulation

26 $$E_{elec}=\sum_{t}\;\sum_{u}Q_{t}^{A}Q_{u}^{B}T_{t,u}$$

27 $$E_{ind}^{A}=-\frac{1}{2}\sum_{t,t^{\prime }}\;\sum_{u,u^{\prime }}\alpha_{t;t^{\prime }}^{A}Q_{u}^{B}Q_{u^{\prime }}^{B}T_{t,u}T_{t^{\prime },u^{\prime }}$$

28 $$E_{ind}^{B}=-\frac{1}{2}\sum_{t,t^{\prime }}\;\sum_{u,u^{\prime }}\alpha_{u;u^{\prime }}^{B}Q_{t}^{A}Q_{t^{\prime }}^{A}T_{t,u}T_{t^{\prime },u^{\prime }}$$

29 $$E_{disp}=-\sum_{t,t^{\prime }}\;\sum_{u,u^{\prime }}D_{t,u;t^{\prime },u^{\prime }}T_{t,u}T_{t^{\prime },u^{\prime }}$$

where $Q_{i}^{A}=\langle p|{\mathrm{Q̂}}_{i}|p\rangle$ and $Q_{i}^{B}=\langle q|{\mathrm{Q̂}}_{i}|q\rangle$ are the expectation value of the corresponding moment operator for the state (Ψ_p_^A^Ψ_q_^B^) of interest, and α represents the polarizability of each molecule

30 $$\begin{array}{l} \alpha_{t;t^{\prime }}^{A}=2\sum_{m\neq p}\frac{Q_{t}^{A\left(pm\right)}Q_{t^{\prime }}^{A\left(mp\right)}}{W_{m}^{A}-W_{p}^{A}}\\ \alpha_{u;u^{\prime }}^{B}=2\sum_{m\neq q}\frac{Q_{u}^{B\left(qm\right)}Q_{u^{\prime }}^{B\left(mq\right)}}{W_{m}^{B}-W_{q}^{B}} \end{array}$$

where $Q_{n}^{A\left(ij\right)}=\langle iq|{\mathrm{Q̂}}_{n}^{A}|jq\rangle$ is the expectation value of the transition moment between the states |*iq*⟩ and |*jq*⟩, and $Q_{n}^{B\left(ij\right)}=\langle pi|{\mathrm{Q̂}}_{n}^{B}|pj\rangle$ is the expectation value of the transition moment between the states |*pi*⟩ and |*pj*⟩. Finally, *D*_t,u;t′,u′_ are the dispersion coefficients

31 $$\begin{array}{ll} D_{t,u;t^{\prime },u^{\prime }} & =\frac{\hbar }{2\pi }\int_{0}^{+\infty }d\omega \alpha_{t;t^{\prime }}^{A}\left(i\omega \right)\alpha_{u;u^{\prime }}^{B}\left(i\omega \right)\\ & -\sum_{n<q}\alpha_{t;t^{\prime }}^{A}\left(W_{n}^{B}-W_{q}^{B}\right)Q_{u}^{B\left(nq\right)}Q_{u^{\prime }}^{B\left(nq\right)}\\ & -\sum_{m<p}\alpha_{u;u^{\prime }}^{B}\left(W_{m}^{A}-W_{p}^{A}\right)Q_{t}^{A\left(mp\right)}Q_{t^{\prime }}^{A\left(mp\right)}\\ & +4\sum_{m<p}\;\sum_{n<q}\frac{Q_{t}^{A\left(mp\right)}Q_{t^{\prime }}^{A\left(mp\right)}Q_{u}^{B\left(nq\right)}Q_{u^{\prime }}^{B\left(nq\right)}}{W_{m}^{A}+W_{n}^{B}-W_{p}^{A}-W_{q}^{B}}. \end{array}$$

The last term in eq 31 contains all possible combinations of multipole transitions from state |*pq*⟩ to all lower energy levels in both molecules, generalizing the standard formulation of the dispersion energy to account for molecules in excited states. When both molecules are in the ground state (*p* = 0 and *q* = 0), all sums ∑_*m*<*p*_ and ∑_*n*<*q*_ disappear, resulting in the classical expression found in books.^45^ When only one molecule is in the ground state (e.g., *q* = 0), then this expression reduces to more advanced formulations found in the literature, like the one given in ref 56.

### 2.3 A System-Specific Representation of LR Intermolecular Interactions

The description of LR interactions provided by eqs 26–29 separates all terms in each expansion into (i) a constant part—involving multipole moment operators, polarizabilities, and dispersion coefficients—i.e., coefficients depending on the symmetry and properties of the molecules’ charge distributions; and (ii) a functional part, given by the *T*-tensors, that describes the dependence with the distance and relative orientation between the molecules. However, if a molecule has symmetry, some of the coefficients will be zero. Only the interaction between two charged molecules with no symmetry (C_1_ symmetry point group) contains all possible terms in eqs 26–29. For any other case, the expansions are simplified in such a way that the resulting expressions reflect the nature of the system. Conceptually in our approach, we start with the expression for the most complex scenario—where both molecules are charged and have *C*_1_ symmetry. Then, the formulas are adapted (pruned) to treat any particular case by removing all terms in the expansion for which the corresponding coefficients are zero.

The point group symmetry of the molecule determines the nonzero components of the multipoles, polarizabilities, and dispersion coefficients, but it is important for a general/practical implementation to adhere to certain conventions. Table 2 lists all of the molecular symmetry point groups: *C*_∞v_, *D*_∞h_, *C*_1_, *C*_s_, *C*_*i*_, *C*_n_, *C*_nh_, *C*_nv_, *D*_n_, *D*_nh_, *D*_nd_, *S*_2n_, *T*, *T*_h_, *T*_d_, *O*, *O*_h_, *I*, *I*_h_, and indicates our convention for how the molecule should be oriented. For each point group in the table, check marks highlight the corresponding subset of generating elements to be considered when placing the molecule into the MF frame: *C*_n_^*z*^ = *n*-fold rotation axis around *z*; *C*_2_^*x*^ = *C*_2_ rotation axis around *x*; σ_*xy*_ and σ_*xz*_ designate *xy* and *xz* as reflection planes; *i* = inversion center at the origin of the MF frame; *S*_n_^*z*^ = *S*_n_ roto-reflection axis around *z*; *C*_3_^*^ = *C*_3_ rotation axis around *x* + *y* + *z* = 1 (for tetrahedral, octahedral and icosahedral symmetries only); *C*_5_^*^ = *C*_5_ rotation axis around $\frac{1}{2}\left(1+\sqrt{5}\right)x+y=0$ (icosahedral symmetry only).

**Table 2:** LRF’s Conventions for Orientation of Molecules of All Symmetry Point Groups with Respect to Relevant Symmetry Elements^a^

	*C*_n_^*z*^	*C*_2_^*x*^	σ_*xy*_	σ_*xz*_	*i*	*S*_n_^*z*^	*C*_3_^*^	*C*_5_^*^
*C*_∞v_	√	–	–	–	–	–	–	–
*D*_∞h_	√	–	√	–	–	–	–	–
*C*_1_	–	–	–	–	–	–	–	–
*C*_s_	–	–	√	–	–	–	–	–
*C*_*i*_	–	–	–	–	√	–	–	–
*C*_n_	√	–	–	–	–	–	–	–
*C*_nh_	√	–	√	–	–	–	–	–
*C*_nv_	√	–	–	√	–	–	–	–
*D*_n_	√	√	–	–	–	–	–	–
*D*_nh_	–	√	–	–	–	√	–	–
*D*_nd_	√	√	–	–	–	√	–	–
*S*_2n_	–	–	–	–	–	√	–	–
*T*	√	–	–	–	–	–	√	–
*T*_h_	√	–	√	–	–	–	√	–
*T*_d_	–	–	–	–	–	√	√	–
*O*	√	–	–	–	–	–	√	–
*O*_h_	√	–	√	–	–	–	√	–
*I*	√	–	–	–	–	–	√	√
*I*_h_	√	–	√	–	–	–	√	√

a Check marks indicate which elements should be present when the molecule is appropriately oriented in the MF frame. See the text for details.

Our procedure to identify the nonzero components in the spherical representation is similar to how character tables often list how vectors transform in the Cartesian representation. For example, consider a molecule with *C*_3v_ symmetry. The multipole moment operators ${\mathrm{Q̂}}_{lm}$ must first be invariant with respect to a 3-fold rotation axis around *z*: ${Ĉ}_{3}^{z}{\mathrm{Q̂}}_{lm}={\mathrm{Q̂}}_{lm}$ , which dictates that

32 $$Y_{lm}\left(\theta ,\varphi +\frac{2\pi }{3}\right)=Y_{lm}\left(\theta ,\varphi \right)\Leftrightarrow e^{im\varphi }=e^{im\left(\varphi +\frac{2\pi }{3}\right)}$$

Therefore, *Q*_lm_ = 0 unless *m* = 3*k* (k∈Z) . In our final notation (cf. eq 26), this means that after considering the *C*_3_ rotation operation, the nonzero coefficients will be {*Q*_00_, *Q*_10_, *Q*_20_, *Q*_30_, *Q*_33*c*_, *Q*_33s_, ··· }.

This process is then extended to other point groups in the *C*_nv_ family: multipole moments *Q*_lm_ must be zero, unless *m* = *nk*

33 $${Ĉ}_{n}^{z}{\mathrm{Q̂}}_{lm}={\mathrm{Q̂}}_{lm}\Leftrightarrow m=nk\left(k\in Z\right)$$

Surviving terms must also satisfy the other operations in the *C*_nv_ group. We have completed the same analysis for all the symmetry operations listed in Table 2 and thus determined the nonzero spherical multipoles for every point group, with the results stored in the LRF code. Once the nonzero multipole moments are known for a given symmetry, they can be used to obtain the nonzero polarizabilities, which in turn can be used to obtain the dispersion coefficients.

Once all the nonzero coefficients in eqs 26–29 are established, we compute the corresponding *T*-tensors by using their recursive relationships—this practical solution to obtain the *T*-tensors in the spherical formulation was introduced by Hättig in 1996.^57^ Upon specification of the system, LRF generates and stores the complete case-specific expansion up to the 15th-order. This takes only a few seconds. For the fitting, at each step, only the specified terms are employed.

### 2.4 Fitting the Expansion Coefficients

After the system-specific full expansion is constructed, fitting the coefficients in this representation is intrinsically nonlinear. Unlike linear fits, nonlinear optimization demands iterative strategies, careful initialization, and is vulnerable to convergence to local minima, making the process increasingly complex as the number of terms grows. The fitting in LRF is performed by the MATLAB fitnlm routine (in the Statistics and Machine Learning Toolbox), which is an implementation of the Levenberg–Marquardt algorithm. LRF’s default tolerances for fitnlm can be adjusted in the advanced fitting options. The initialization takes advantage of system specifications, such as charge, symmetry, and whether the two molecules are identical or chiral partners, to constrain the coefficients. The user can—but is not required to—input any known quantities, either as fixed values or as initial values for the fitting. By default, the initialization routine also looks at a portion of the data set furthest into the long range and does a preliminary fit to the coefficients from the first two leading orders, estimating also the asymptotic energy. In LRF, the user does not need to “zero” the data set; rather, LRF finds the best fit for the asymptote as one of the coefficients. Once initialized, in our experience, the most robust way to proceed toward a satisfactory fit is to introduce the terms of the expansion, order by order, updating the fit at each step, monitoring the behavior and statistics of the fit, until sufficient accuracy is achieved.

The dimensionality of the PESs treated by LRF ranges from one (atom–atom) to six (two nonlinear molecules). For construction of PESs describing the close interaction region, the difficulty of achieving an accurate fit, and the amount of ab initio data required, usually scales strongly with dimension. In contrast, for representation of the long range, the amount of data required is mainly dictated by the number of parameters in the expansion and is less closely tied to dimensionality. Upon specification of the system, charges, symmetry, identical or different molecules, and the order of the expansion, LRF will determine the number of parameters. The fitting data set should then be computed to sample at least the minimal symmetry subspace of angles and range of *R* and be generous with respect to the number of parameters to be fitted (ca. 5–10 times larger) but generally requires far fewer points than the close interaction region. The fitting itself takes between a few seconds and a few minutes. This depends on the number of ab initio data points and the number of coefficients (which scales with the order of the expansion). For the 4D example shown in Figure 6, which includes an overly generous set of 4000 data points, fitting the first few orders using LRF on a laptop PC takes only a few seconds. The time-to-solution increases as higher terms are added, reaching 2–3 min for the eighth-order expression.

## 3 LRF: Features and Capabilities

LRF applies the methodology outlined above for systems in nondegenerate states by implementing the analytic long-range expansions (up to 15th-order) via a tensorial spherical basis with recursive relations. Symmetry, fragment charges, and intermolecular relationships are automatically accounted for, providing a symmetry-adapted expansion for any pair of neutral or charged—identical, chiral, or distinct—partners. The entire procedure is controlled through a Windows MATLAB-based graphic user interface (GUI). The interface gives users control over the fitting model and constraints, includes diagnostics to assess fit quality, flag anomalies in the ab initio data, and provides 1D/2D visualizations of the—electrostatic, induction, or dispersion—interactions with respect to the various coordinates. LRF provides an accurate representation of the long-range PES, and it is simple to use even for nonspecialists.

As can be seen in the program workflow, shown in Figure 3, the process begins with (step 1) the “system definition”, specifying each molecule by symmetry group and net charge; and (step 2) uploading an input file (if any) containing the ab initio data to be used in the fit. There are no special requirements regarding the number of data points or their distribution. This is all done in the “SETUP” tab (see Figure 4). Relevant information concerning the input file (type of file, structure, choices of energy and coordinate units of the input data, and more) can be found in the Supporting Information. Once the initialization is complete, tables of the relevant interaction terms organized by order can be consulted in the “EXPANSION” tab (see Figure 5). This valuable information is available after the symmetry of the molecules is specified, even if no fit is to take place. Finally, in the “FITTING” tab (see Figure 6), the user can (step 3) select which interaction terms to include and fit the coefficients, and once the fit has converged to the desired accuracy (step 4) export the coefficients.

**Figure 3:**
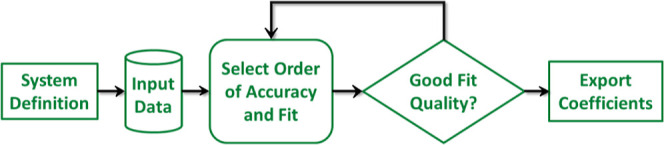
LRF flowchart for construction of the long-range representation.

**Figure 4:**
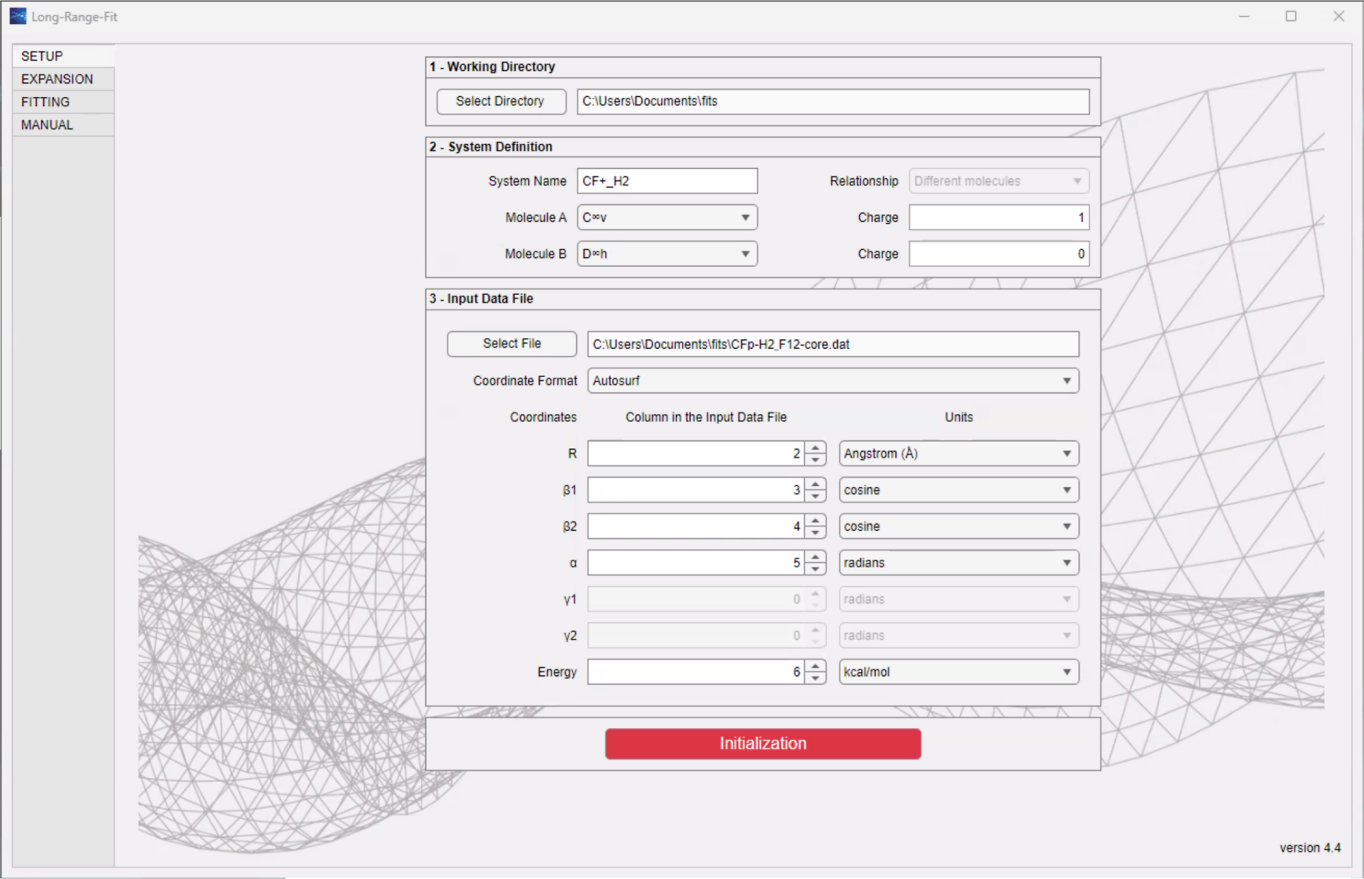
The “SETUP” tab is employed for the first two steps in the flowchart: define the system and initialize the program.

**Figure 5:**
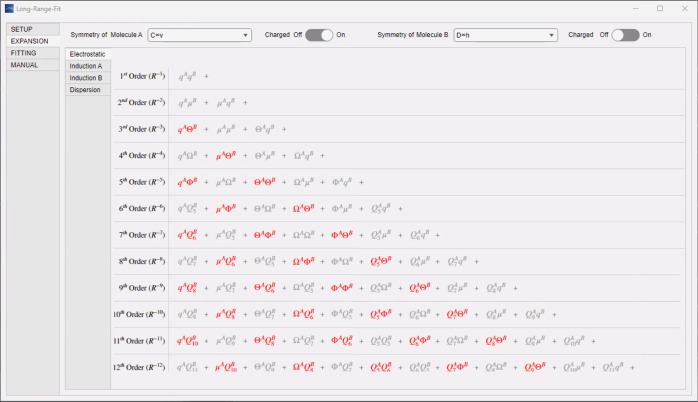
The “EXPANSION” tab renders tables highlighting the active interaction terms for any specified system. Here, for CF^+^–H_2_, the electrostatics table was selected, and the relevant terms are seen highlighted in red, beginning at third-order. Analogous tables can be rendered for induction or dispersion by selecting them from the menu in the top left of the figure.

**Figure 6:**
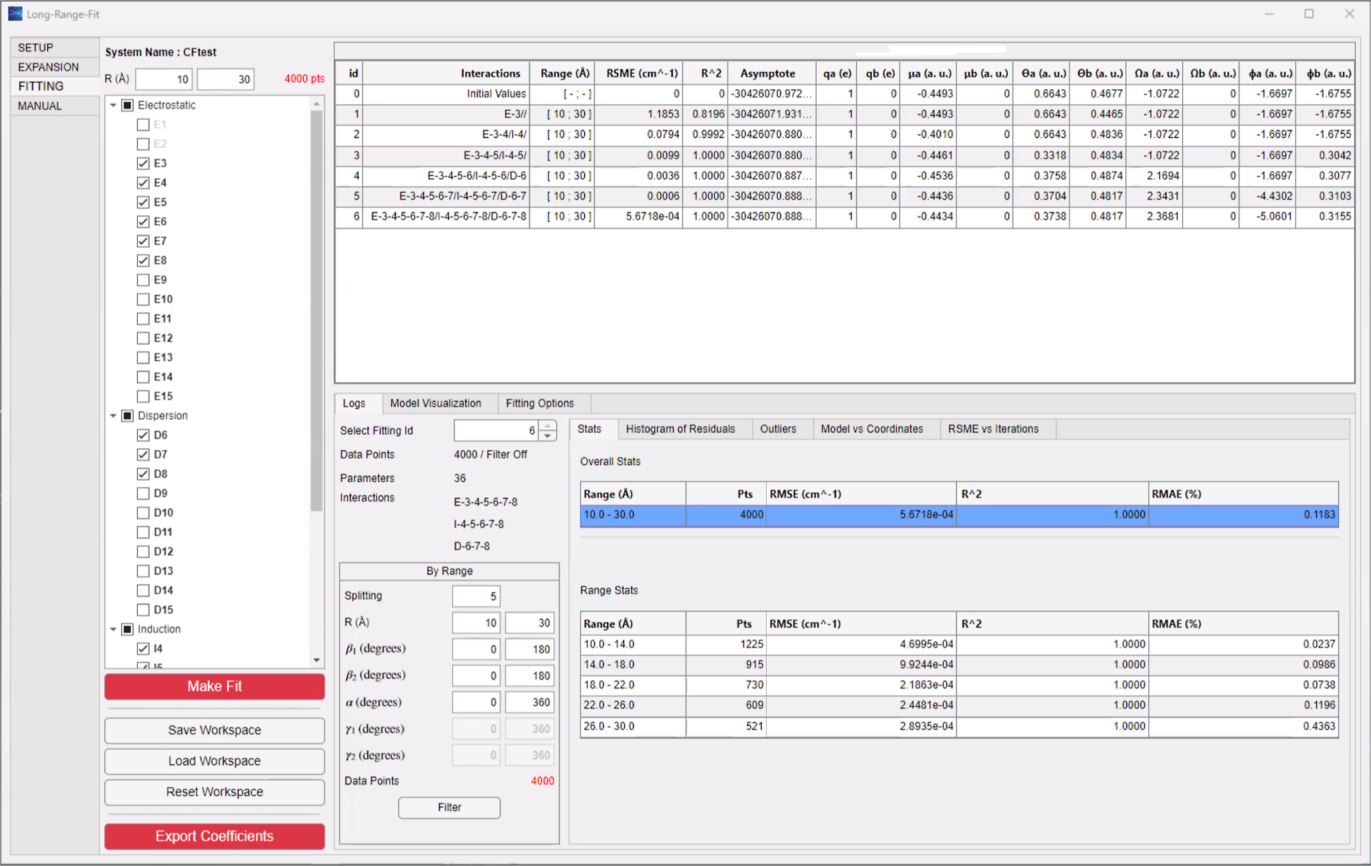
Once the system is defined and the data file is read, the app leads the user to the “FITTING” tab, where the data can be fitted and the coefficients exported once the desired accuracy is reached. The table in the upper-right side follows the progression of an LRF fit to an ab initio data set for the CF^+^–H_2_ system. The fit is performed sequentially, where each row in the table introduces a higher maximum order in the interaction expressions (third through eighth), including all relevant terms from the electrostatic, induction, and dispersion expansions. The fitting statistics improve rapidly and then plateau (see the text).

The LRF MATLAB-based GUI is freely available from the authors for noncommercial purposes. Although based on MATLAB, the use of LRF does not require the installation of MATLAB or a MATLAB license. Only a freely available MATLAB Runtime library is needed (version 2024a for WINDOWS), which is downloaded and installed by the LRF installer. More details about obtaining and using LRF can be found in the Supporting Information.

In the rest of this section, we demonstrate some of the main features and capabilities of LRF by using the software to study the LR interactions for some illustrative examples.

### 3.1 CF^+^–H_2_

Although fluorine is one of the most reactive elements in the ISM, the dominant F-bearing species (HF and CF^+^) are governed by a compact set of reactions whose rates are largely controlled by the local abundances of atomic F, H_2,_ and C^+^.^58,59^ In this context, the CF^+^ and H_2_ molecules are considered crucial species for monitoring fluorine chemistry, as their relatively simple formation pathway makes them promising tracer of atomic fluorine in diverse ISM environments. Our previously reported PES for this system^60^ was constructed to support scattering dynamics calculations relevant to fluorine chemistry in the ISM and includes an analytic LR.

Fitting the LR of this system helps to illustrate several aspects of our approach. Here, we show how upon setup and specifying the fragment charges and symmetries, LRF produces tables of the interaction terms organized by order (Figure 5). Our usual practice is then to introduce the interaction terms, order by order, updating the fit at each step, simply by selecting the desired order of each interaction type in the fitting menu. Figure 6 shows a table that is produced, providing some details of the fit and the derived coefficients. Each fit to a successively higher order adds a row to the table, where the root-mean-square error (RMSE) and the coefficient of determination *R* ^2^ are reported along with the first few multipole coefficients. Statistics of the fit are produced, which help the user determine when a satisfactory fit has been obtained. Figure 7 shows examples of the plots of histograms of fitting residuals, making it easy to see when an accurate balanced fit has been obtained. Once an acceptable fit has been obtained, a complete set of coefficients is exported, formatted to use with an LRF external subroutine to evaluate the PES representation.

**Figure 7:**
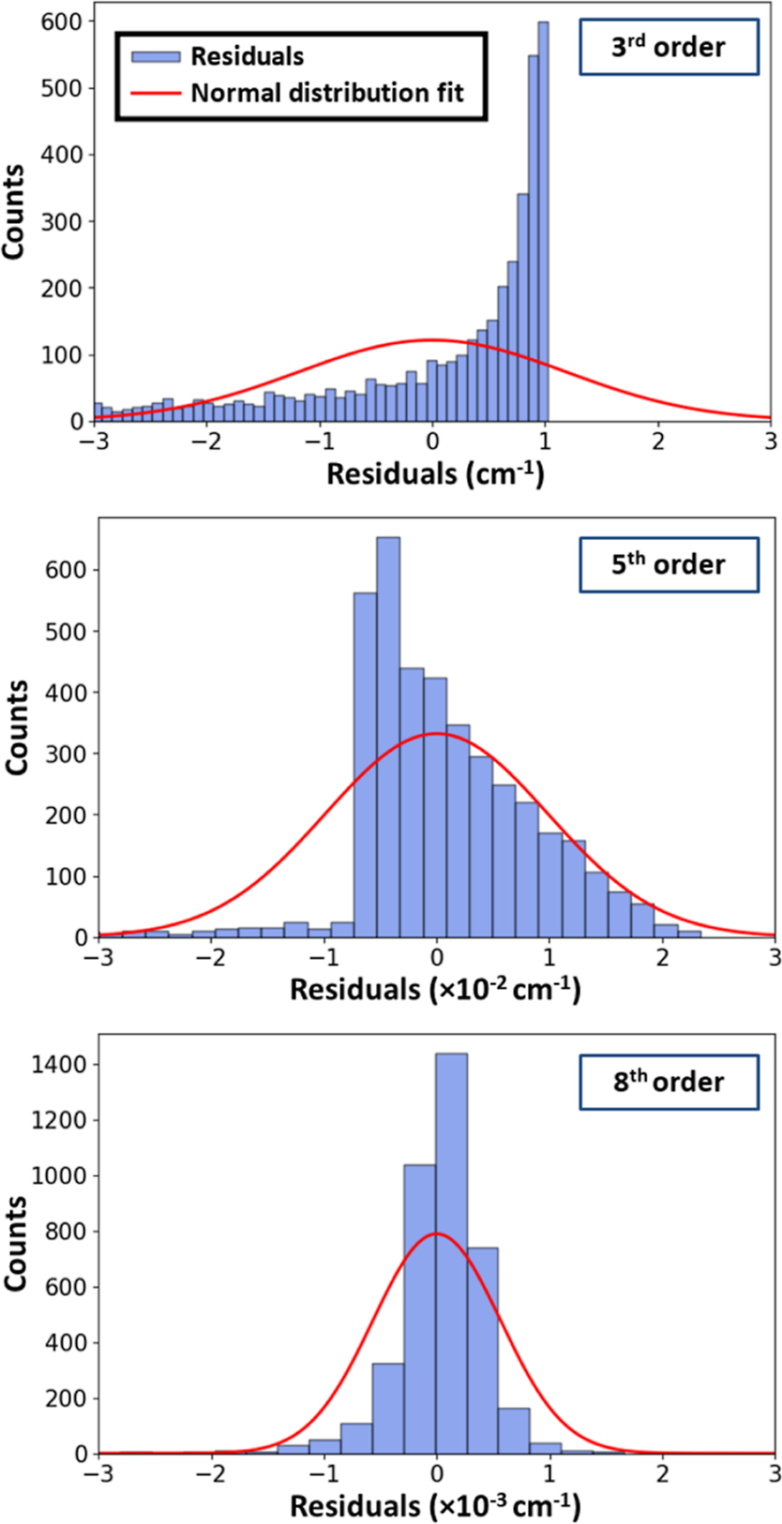
Histograms of fitting residuals are produced by LRF as each order of interaction terms is introduced. Here, for CF^+^–H_2_, the results are plotted for the third-, fifth-, and eighth-order fits (upper, middle, and lower panels, respectively). Using only the leading third-order charge–dipole term, the residuals are large and unbalanced. When sufficient terms are added, they become orders of magnitude smaller and balanced (see the text).

As we reported previously for this system,^60^ the PES reflects remarkably significant angular dependencies far into the LR. Even at *R* ≥ 25 Å, where the interaction is dominated by the electrostatic charge–quadrupole term *q* ^A^Θ^B^ ∝ *R* ^–3^ (see Figure 5) and the induction leading term ${\left(q^{A}\right)}^{2}\alpha_{\mu \mu }^{B}\propto R^{-4}$ , there are still energy variations of nearly 2 cm^–1^ with respect to the orientation of H_2_, and moving to shorter distances, these variations become rapidly larger. Moreover, even at longer distances, employing only the leading terms is not sufficient to accurately fit the ab initio data. This is reflected in both the fitting error (see the table in Figure 6, second row) and the observation of an unbalanced residual distribution (see Figure 7, upper panel) produced by error analysis tools implemented in the LRF code.

When an insufficient number of terms are employed in the fitting expansion, the coefficients for those few leading terms will deviate substantially from physically realistic values—in order to best accommodate the data—and lacking the correct angular and radial dependencies, the fit will have relatively large and unbalanced errors. A systematically nonrandom distribution of residuals reflects an inadequate model.^61^ On the other hand, if one fixes the coefficients for only a few leading terms to known benchmarks (either from experiment or theory), but excludes other relevant terms, then again the detailed topography of accurate energy data will not be well reflected.

Note also that when fitting the set of interaction terms, there may be ambiguities. For example, if one fits a data set of interaction energies between two different molecules A and B, using only the dipole–dipole term, expressed as *E*_3_ = (μ_10_^A^μ_10_^B^)*T*_10,10_, then the derived coefficient is the product μ_10_^A^μ_10_^B^ of the two dipoles, with no information about the separate magnitudes. Even if the two molecules are identical, there is still a sign ambiguity. However, if even one coefficient is known (such as the charge of fragment A in the CF^+^–H_2_ system), then a cascade of determinations occurs through the set of coefficients. Here, the known charge value determines the related multipoles in the electrostatic expansion, such as Θ_20_^B^ in the leading term *q* ^A^Θ_20_^B^, which in turn defines others, until all included multipoles are determined. Once the multipoles are known, the polarizability coefficients become determinable in the induction terms, which also define the dispersion coefficients. A similar effect can be achieved by knowing and fixing at least one coefficient in neutral systems, which is convenient since often at least one quantity might be available or easily computed.

For the CF^+^–H_2_ system, our data set for the PES was generated at the CCSD­(T)-F12b/CBS level, with all electrons included in the correlation treatment, which is generally spectroscopically accurate in light-atom systems where affordable. To estimate the CBS limit, total energy data from the CVTZ-F12 and CVQZ-F12 basis sets were extrapolated using the *l* ^–3^ formula.^62^ At this high level of electronic structure theory, one expects physically realistic coefficients from the fitting, though generally some differences can be expected due to basis set incompleteness, order of correlation treatment, inclusion/exclusion of other corrections, truncation of the fitting expansion, and fitting error. The data span 10–30 Å of separation, and with only the leading third-order interaction (electrostatic charge–quadrupole), the RMSE is large, about 1.2 cm^–1^ and the *R* ^2^ is only 0.8. The histogram of residuals shown in the first panel of Figure 7 is very unbalanced, and the fit is deemed poor. At fourth-order (third row in the table, Figure 6), adding the dipole–quadrupole and introducing the first induction term dramatically improve the fitting error and *R* ^2^ to 0.08 cm^–1^ and 0.999, respectively. Induction seems quite important, and by fifth-order, the overall fit is already quite good. Dispersion enters at sixth-order, but its inclusion appears to be less important. The fitting error improves by roughly an order of magnitude with each increase in the order of the fit, before plateauing between seventh- and eighth-orders. No significant further improvements were found in tests up to 12th-order. The hexadecapole of H_2_ first enters at fifth-order (row 4 in the table, Figure 6) and its coefficient can be seen to jump to a realistic value in that row, from being essentially undetermined at lower orders. As seen in the table, the coefficients adjust slightly as new terms are introduced but are relatively stable. The final RMSE of 0.0005 cm^–1^ is on the order of the convergence of the electronic structure data and together with the balanced histogram of residuals is deemed to be a practically perfect fit. We interpret the abrupt plateau in the convergence behavior to indicate that at that order, all of the relevant terms needed to represent the coordinate range in the data set are included. If the data were extended to shorter interaction distances, then higher-order terms would become valuable/necessary.

For this system, it is remarkable how accurately the known quantities of H_2_ were obtained, incidental to our fitting. A 2018 paper by Miliordos and Hunt^63^ studied the bond distance dependence of electrostatic and polarizability expansions of H_2_ at the CCSD level, which is Full-CI for isolated H_2_. Their results are consistent with previous high-level studies of H_2_. In table V of their paper, they list their best computed values for the quadrupole, hexadecapole, and both components of the dipole-polarizability tensor for *r*_H_2__ = *r*_0_. For the quadrupole and hexadecapole, our values from fitting the LR of the CF^+^–H_2_ data set are 0.4817 and 0.3155 au, respectively, extremely close to the values of 0.4823 and 0.3139 au reported by Miliordos and Hunt. For dipole polarizability, they report the two Cartesian tensor components α_*zz*_ and α_*xx*_ at 6.7178 and 4.7308 au, respectively. These values can be directly compared to the two components of our spherical tensor representation, α_10,10_ and α_1*c*,1*c,*_ for which we obtain values of 6.7023 and 4.5633 au, respectively.

### 3.2 C_10_H^–^–H_2_

Carbon-chain anions have been identified in interstellar and circumstellar media fairly recently, offering insights into the physical and chemical conditions in different astrophysical contexts.^64–66^ Their quantities also inform the free electron density, which relate to the rate of cloud collapse and star formation. Carbon-chain anions are much less abundant than their neutral counterparts, and current chemical models fail to accurately replicate the observed anion-to-neutral ratios. In cool, low-density areas where anions are found, thermal equilibrium statistics generally do not hold, and accurate emission line modeling must consider radiative and collisional rates with H_2_, the most commonly encountered molecular species.^67,68^

This system, shown in Figure 8, is composed of two linear molecules: the anion C_10_H^–^ (with symmetry *C*_∞v_) and H_2_, which is a neutral linear molecule (with symmetry *D*_∞h_). Since the fragment symmetries and the presence of a charge (negative in this case) are the same as the previous example, we expect to see the same set of interaction types, beginning with the electrostatic charge–quadrupole term (∝*R* ^–3^) and the induction leading term (∝*R* ^–4^). The anion is a long chain of 11 atoms, making the PES extremely anisotropic. However, the anisotropy is more complicated and interesting than just the obvious steric effect of the long C_10_H^–^ fragment but can be interpreted via the leading electrostatic terms. Figure 9 shows radial cuts through the PES for various orientations of the two fragments. The plot also includes the close interaction region, combining two fits into a global representation. The electronic structure data set for this PES was computed at the CCSD­(T)-F12b/VTZ-F12 level. Anisotropy in this context is defined as a strong sensitivity to angular coordinates. For this system, far into the LR, the charge–quadrupole term is dominant and thus the PES has little dependence on the orientation of the anion but depends strongly on the orientation of H_2_ (which governs the quadrupole term). Moving to closer distances, the PES also takes on a strong dependence on the orientation of the C_10_H^–^ anion, first partly due to its large dipole moment and then due also to the steric effect. The shape of C_10_H^–^ dictates that the onset of short-range repulsion occurs much further out for approach to the ends of the molecule, relative to approaching from the side. Indeed, in this way, the boundary defining the onset of the long range by a negligible charge overlap depends on the orientation of C_10_H^–^. Figure 9 shows that when C_10_H^–^ has a side-on orientation (β_A_ = 90), curves for various orientations of H_2_ (β_B_) all have repulsive walls at much closer distances. However, it is fascinating to see that even in the regions of deepest attraction, electrostatic effects are retained such that the PES is enormously sensitive to the orientation of H_2_. For example, around *R* = 9 Å, the collinear (β_A_ = 180, β_B_ = 0) global minimum is located with a well depth of more than 600 cm^–1^, but by only rotating H_2_ from end-on to side-on (β_B_ = 90), the well is eliminated almost completely, becoming more than 500 cm^–1^ less stable (compare the black dashed and light blue traces in the figure). At even closer distances, similar behavior is noted for the cuts with minima around *R* = 3.5 Å, representing the approach to the side of the anion. Thus, overall, the PES has strong sensitivity to the orientation of H_2_, all the way from the far long range to the close interaction regions.

**Figure 8:**
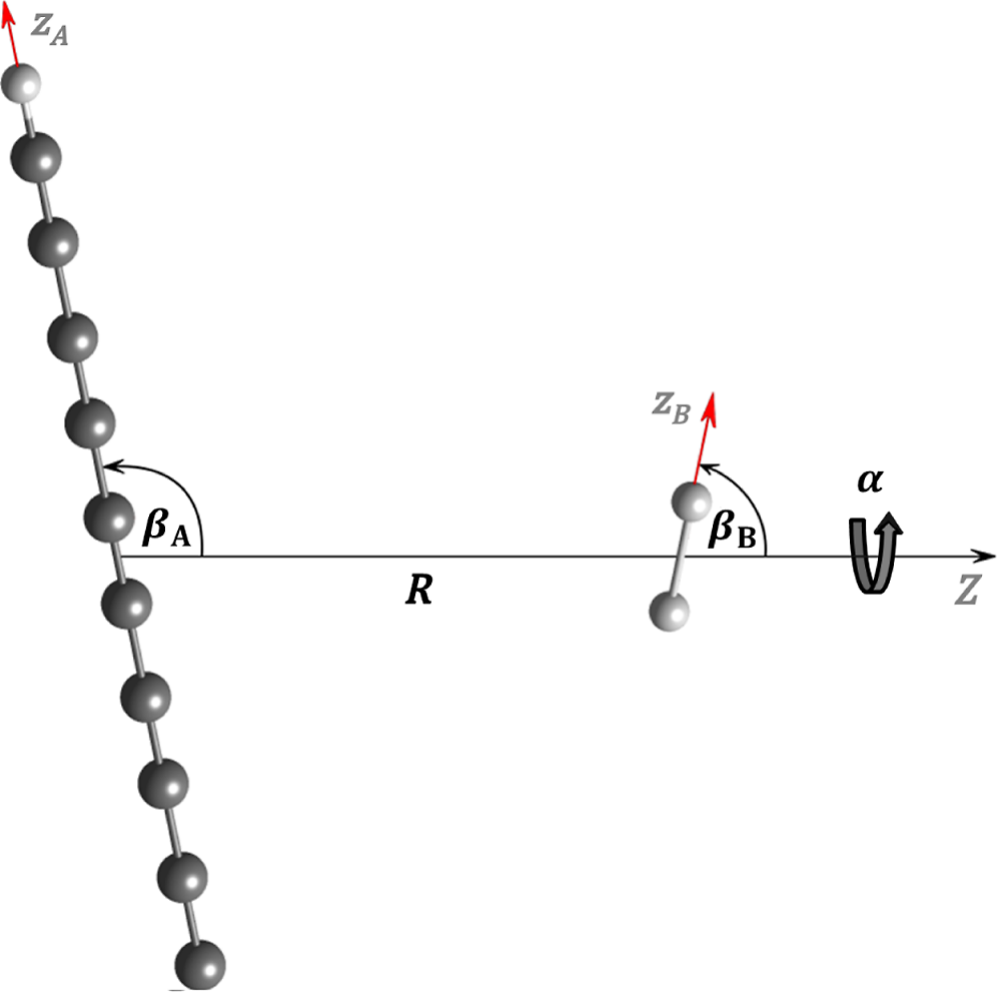
Representation of the internal coordinates for the C_10_H^–^–H_2_ system.

**Figure 9:**
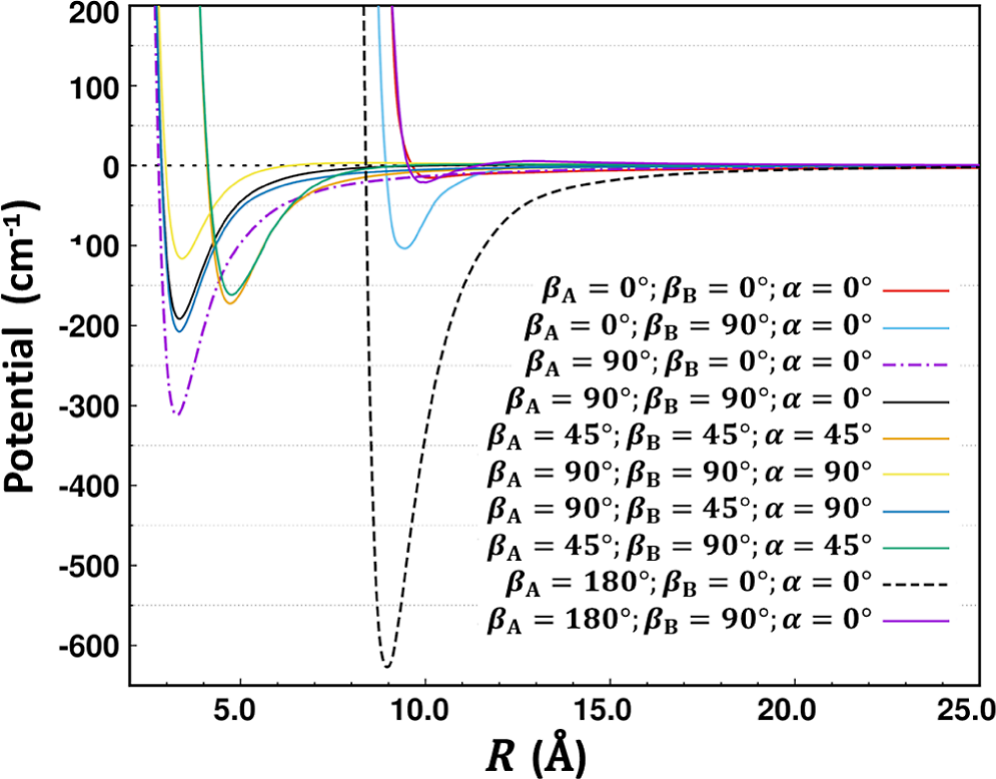
Radial cuts through a combined close interaction/LR PES for C_10_H^–^–H_2_ are plotted for various orientations of the two fragments. Behavior with respect to the center-of-mass distance *R* is shown in the plot. The angles listed in the figure legend in degrees are β_A_, β_B_, and α, respectively, which are the angles shown in Figure 8. Remarkable behavior is noted with respect to the orientation of both C_10_H^–^ and H_2_ (β_A_ and β_B_); see the text.

The effect of the shape of molecules such as C_10_H^–^ on where the onset of the long range can be defined has motivated developments such as distributed multipoles.^69^ Here, we maintain a single expansion but have implemented a strategy to allow some of the data from approach to the side of C_10_H^–^ to be included, since it still meets the negligible overlap criterion. LRF includes an optional “LeRoy Filter” algorithm, with a specified distance parameter, that determines whether a particular geometrical configuration belongs to the fitting set or not, based on the minimal distance for any pair of atoms belonging to different molecules (*r*_*ij*_^min^). This simple approach captures the steric aspects of the charge distribution, allowing classification of the boundary regions. This methodology allows for a more robust fit in cases of large anisotropy since more data points can be included (see Figure 10), providing broader coverage and a smoother switch to the short-range representation. C_10_H^–^–H_2_ is provided as an example system in Supporting Information, including instructions on how to employ this LeRoy Filter algorithm.

**Figure 10:**
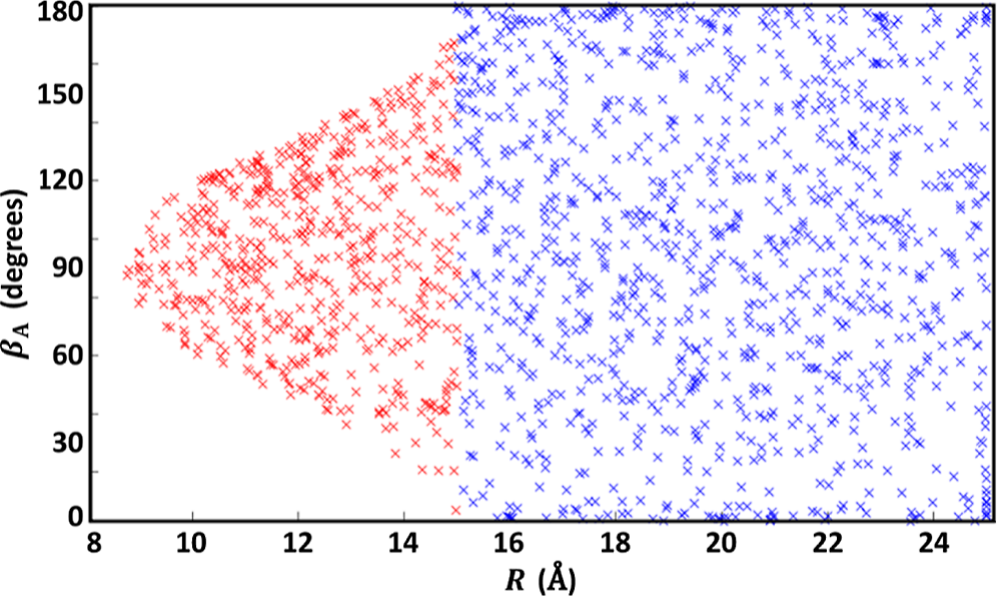
Points from the C_10_H^–^–H_2_ data set are plotted, indicating intermolecular separation *R* and orientation of C_10_H^–^ (β_A_). The plot illustrates the idea behind LRF’s algorithm for treating highly anisotropic data sets (see the text). The method carves out a region (red points), which can be added to what is normally considered the long range beyond a simple distance cutoff (blue points). A hypothetical fixed long-range cutoff at *R*_LR_ = 15 Å partitions the data into *R* < 15 Å (red, ≈35% of all points) and *R* ≥ 15 Å (blue).

C_10_H^–^–H_2_ has the same partner (H_2_) as the previous CF^+^–H_2_ system, but it is much more challenging to represent C_10_H^–^ as a single expansion than it is for CF^+^. Results for the fitting are shown in Figure S3 of Supporting Information. In contrast to the behavior of the CF^+^–H_2_ fit, the fitting error for C_10_H^–^–H_2_ drops more slowly with order, reaching 0.1 cm^–1^ at the 10th-order.

Another important feature is outlier detection. Data sets obtained from ab initio calculations may occasionally contain unconverged points inconsistent with the rest of the data set. Wild errors completely outside the energy range are easily identified, but subtle irregularities can appear with magnitudes of only fractions of a cm^–1^. For example, in some cases, such small disruptions have been attributed to the use of incomplete auxiliary bases in explicitly correlated (F12) calculations. Small errors are harder to identify, especially if the PES includes both attractive and repulsive energies for various combinations of coordinates. LRF identifies outliers to the fit with a weighting that emphasizes relative error, thus becoming more sensitive at large *R*. Any outliers that are not accommodated by a generous set of physically correct interaction terms can be selected for removal. No such points were noted in the example systems discussed here, except in some preliminary data for C_20_H_20_–Ne, as discussed in that section.

### 3.3 CO-Dimer

LRF handles identical-monomer dimers by enforcing exchange symmetry in its angular basis so that the fit is invariant to the exchange of molecules, and the two sets of expansion coefficients are constrained to be equal. (CO)_2_ is one of many examples of such systems. CO is a common species in cometary comae, produced by sublimation of its ice as the comet passes near a star, and therefore, scattering data for CO with a variety of collision partners—other common components of the comae—are needed for modeling those environments. Other components to be considered as collision partners include water, CO_2_, and HCN/HNC, but collisions with other CO molecules are also important to model.^70–75^

The nature of LR interactions in (CO)_2_ helps to illustrate the strength of our LRF method and emphasizes some of the points we have been making about the possible inadequacy of a few leading terms to represent the long range. CO is a polar molecule, so the leading electrostatic term is the dipole–dipole interaction, proportional to *R* ^–3^, appearing three orders before the induction and dispersion interactions which both enter at sixth-order and are proportional to *R* ^–6^. The dipole of CO is known to be small (0.043 au) in the ground vibrational state and, in an interesting contradiction to expectations based on atomic electronegativities, is oriented with the negative end on the C atom. Our ab initio data set is again at the CCSD­(T)-F12b/CBS level with all electrons correlated and was used to construct a PES of demonstrated spectroscopic accuracy^70^ and so is expected to accurately reflect the electronic structure. Fitting the data set in the distance range of 6–30 Å finds the single leading dipole–dipole term almost useless, yielding an RMSE of 3.9 cm^–1^ and an *R* ^2^ of only 0.07, with the resulting model qualitatively different from the PES. Adding the fourth- and fifth-order electrostatics, which includes the quadrupole–quadrupole interaction among other terms, yields only a modest improvement with values of 3.3 cm^–1^ and 0.35 for the RMSE and *R* ^2^, respectively. The fit quality changes dramatically at the sixth-order. Adding the sixth-order electrostatic terms alone does not produce any improvement, but with the sixth-order induction and dispersion included, the fitting error improves by an order of magnitude, and the *R* ^2^ jumps above 0.99. At this stage, the model is in qualitative agreement with the ab initio data, though still higher terms do continue to improve the fit, which reaches an RMSE of 0.01 cm^–1^ at 12th-order. The coefficient for the dipole, obtained by fitting the data as an LR expansion is 0.094 au, small as expected, but somewhat larger than the experimental value (0.043 au). To explore this difference a bit further, we performed a four-point finite field calculation of the monomer dipole at the same level of electronic structure theory as the PES (CCSD­(T)-F12b/CBS), which yielded a result of 0.045 au, very close to the experimental value. The CO-dimer system has long been known to be significantly impacted by even higher-order correlation not captured by CCSD­(T).^76,77^ Thus, our interpretation of the fitting behavior for this case is that while the monomer dipole is well captured at the employed level of theory, higher terms in the interaction are less well represented and the dipole coefficient adjusts slightly to compensate for other terms in order to minimize the fitting error.

Figures 11 and 12 illustrate the behavior of the fits and the relevance of various contributions. In Figure 11, the combined electrostatic terms from third- to sixth-order are compared with the total energy for distances beyond 16 Å. As seen in the figure, the electrostatic contribution matches the total energy quite well in the far-range, and the two merge near 25 Å. In contrast, for shorter distances, as seen in Figure 12, the combined electrostatic terms from third- to sixth-order simply lack the necessary form (both radial and angular) to represent the data and gross errors are seen in comparison to the total energy. This highlights the key role played by induction/dispersion in this system and the importance of including a sufficient set of interactions to obtain an accurate and balanced representation.

**Figure 11:**
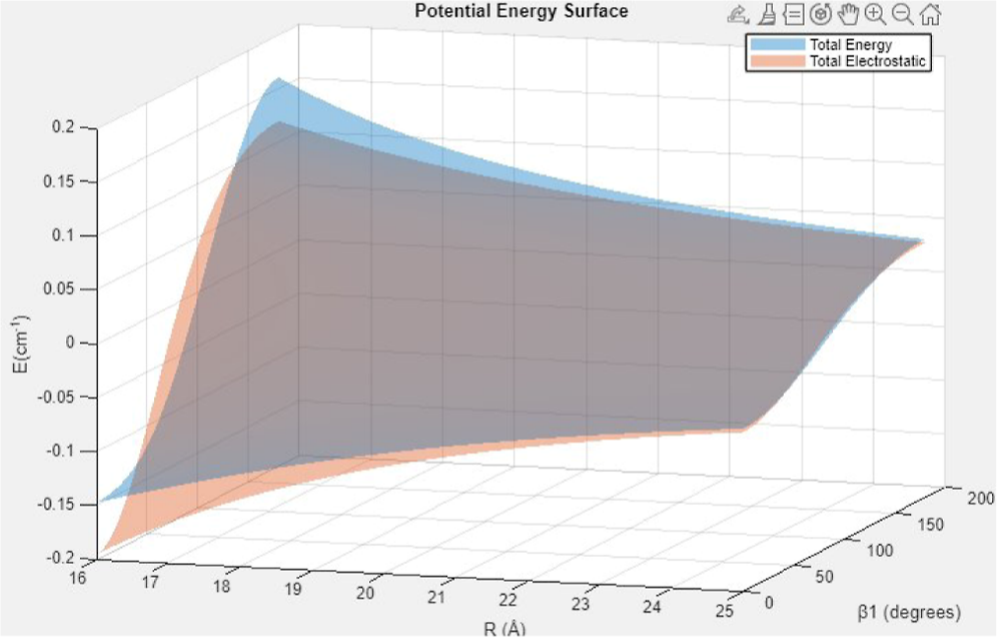
CO–CO long-range potential: total energy (blue) and electrostatic contribution (orange) as functions of separation *R* and orientation β_1_. Angles α and β_2_ are both fixed at 0 degrees. In the *R* = 16–26 Å regime, the interaction energy is small and the residual anisotropy is governed by the *R* ^–3^ dipole–dipole and *R* ^–4^ dipole–quadrupole terms, while induction/dispersion (∝*R* ^–6^) becomes negligible. The decomposition highlights that far-field behavior is dominated by electrostatics (see the text).

**Figure 12:**
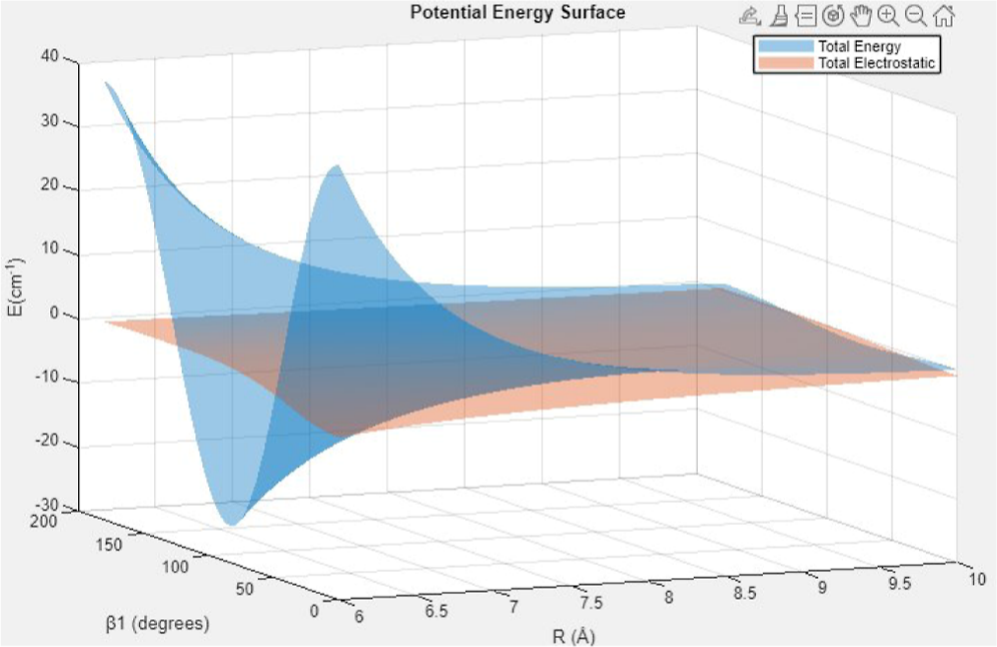
CO–CO midrange potential: total energy (blue) and electrostatic contribution (orange) versus *R* and β_1_ for *R* = 6–10 Å. Despite electrostatics beginning at third-order, the well depth and strong orientational dependence arise primarily from induction and dispersion. The gap between the two surfaces highlights the dominance of nonelectrostatic interactions in the near field (see the text).

### 3.4 C_20_H_20_–Ne

Fascination with highly symmetric systems goes back at least as far as Plato’s dialogues, and society as a whole has shown special interest in molecular manifestations of the Platonic solids such as tetrahedrane, cubane, octahedrane, dodecahedrane, and of course fullerene. Scientists are studying such systems not only for reasons such as their synthesis and reactivity, but also for their possible unusual behavior and properties, as hosts of encapsulated molecules, for cold control, and even as possible molecular qubits.^78–80^

Symmetry has strong implications for LR interactions. For example, as we mentioned in the Introduction, the leading electrostatic term for interactions between two C_60_ fullerenes is at 13th-order, while dispersion begins at sixth. The efficiency of cooling rotational degrees of freedom via collisions, e.g., the rate of rotationally inelastic collisions, is largely determined by the anisotropy of the PES. Although dispersion begins at the sixth-order, it is higher-order dispersion terms that provide anisotropy, the subtle corrugation of the PES for these systems. This is why rotationally cooling C_60_ by collisions with atomic bath gases like He or Ne has proven to be so inefficient.^81^

Studying such systems computationally also presents challenges. Dodecahedrane and C_60_ have 140 and 360 electrons, respectively, even without a collision partner, making them prohibitively expensive to treat with large basis sets and high-order correlation treatments in supersystem calculations. On the other hand, trying to estimate the interactions from a monomer property-based approach is also difficult given the high order of the relevant terms.

Here, as an illustration, we surveyed the LR interactions of dodecahedrane (C_20_H_20_) with a neon atom (Ne). First, we computed a small data set of interaction energies distributed in distance from *R* = 6–30 Å using a new local correlation method called PNO-LCCSD­(T)-F12 as implemented in MOLPRO 2025.2, which has been introduced as a cost-effective beyond-DFT approximation to explicitly correlated coupled-cluster theory.^82,83^ We employed recommended tight settings of DOMOPT = TIGHT, DOMOPT_INTMOL = TIGHT+,^83^ but noted some behavior in the data that made it unsuitable for this purpose. We observed a small discontinuity (17 cm^–1^) in a radial cut, just past 10.5 Å, and also some general noise throughout (e.g., erratic points along angular test cuts), on the order of a few wavenumbers. This is likely due to the domain approximations in the local correlation method. The tightness of the domain approximations for PNO-LCCSD­(T)-F12 significantly affects the cost, so we did not tighten them further since the cost for each geometry was already quite high, and the goal here was to illustrate capabilities of LRF, rather than performing a high-level study of dodecahedrane. Though perhaps less accurate, we switched to the DF-MP2-F12 method, which is not a local correlation method, in order to get more precisely converged data to better determine the subtle anisotropy of this system. Ultimately, about 170 energies were computed using MOLPRO at the DF-MP2-F12/VDZ-F12 level, each taking about 2 h to converge using 4 cores. This relatively small number of points is generous due to the very high symmetry, which dictates that there are only a few nonzero coefficients.

Dodecahedrane has icosahedral symmetry, and Ne has spherical symmetry, which significantly limits the number of terms in the LR expansion. For the interaction with Ne, there are no electrostatic or induction terms through the 15th-order, but dispersion appears in even numbered orders beginning at the sixth (sixth, eighth, 10th, ...). The sixth- and eighth-order contributions are isotropic, so anisotropy enters at 10th-order, which limits its effect to relatively close distances as discussed also by Klos et al. for C_60_.^81^

The interaction between C_20_H_20_ and Ne is perhaps counterintuitive, considering the enormous size of C_20_H_20_, but can be rationalized based on the high-order nonzero terms in the LR expansion. The system is shown in Figure 13, with the H atoms extending slightly more than 3 Å in all directions from the origin at the center-of-mass. Due to the high symmetry of C_20_H_20_, and the spherical collision partner, there is very little interaction at longer distances. As shown in Figure 14, the interaction, governed by the even ordered dispersion series, turns on significantly inside of 7.5 Å, which given the size of C_20_H_20_ is quite close. In our radial test cuts with the DF-MP2-F12 method, we found the minimum to be near 6 Å, and further in, at 5 Å, is already significantly repulsive (above the asymptote). The anisotropy is also apparent in the plot as waviness of the lines. As mentioned above, anisotropy begins with the 10th-order term in the dispersion series, which greatly limits the range of its relevance. The symmetry and anisotropy can be better appreciated in Figure 15, which plots the PES with respect to the two angles with *R* fixed at 7.0 Å. There is 4.7 cm^–1^ of variation in Figure 15, where the mean value is −29 cm^–1^, so the relative anisotropy is about 16%. This is in contrast to the nature of the C_60_ with He or Ar systems mentioned earlier,^81^ where the anisotropy in the long range was mentioned to be only about 1% and would correspond to extremely low efficiency for collisionally cooling the rotational temperature. Note, however, that the relative anisotropy is strongly dependent on distance. In our results, the relative anisotropy drops off rapidly to only 0.16% at *R* = 12 Å, a 100-fold decrease. He and Ne are the most commonly employed cooling buffer gases in experimental studies. Those authors mention that for C_60_, anisotropy does increase in the close interaction region due to nondispersive contributions, and similar effects are expected for C_20_H_20_ also. We ascribe the markedly more significant anisotropy in the LR of C_20_H_20_ vs C_60_, being due to the hydrogen atom sticking out from each C atom in C_20_H_20_. Thus, even with the same high *I*_h_ symmetry, the anisotropy can vary significantly, but since the anisotropy is governed by terms starting at the 10th-order, it is quite constrained in terms of the range of distances for which it is relevant. In our results, the relative anisotropy for C_20_H_20_ and Ne drops off at a rate with an R-dependence between 1/*R* ^7^ and 1/*R* ^8^, but this is interpreted with caution due to the modest level of theory and small basis set. Lowering the symmetry of the system of interest via functionalization, or perhaps that of the buffer gas, can change the nature of the interactions dramatically and would affect the efficiency of cooling. No doubt, there are practical constraints on the choice of buffer gases, but it is interesting to note that (without modifying the system of interest) switching to a molecular collider—even H_2_—would turn on lower-order terms with anisotropy, including those from the electrostatic and induction series.

**Figure 13:**
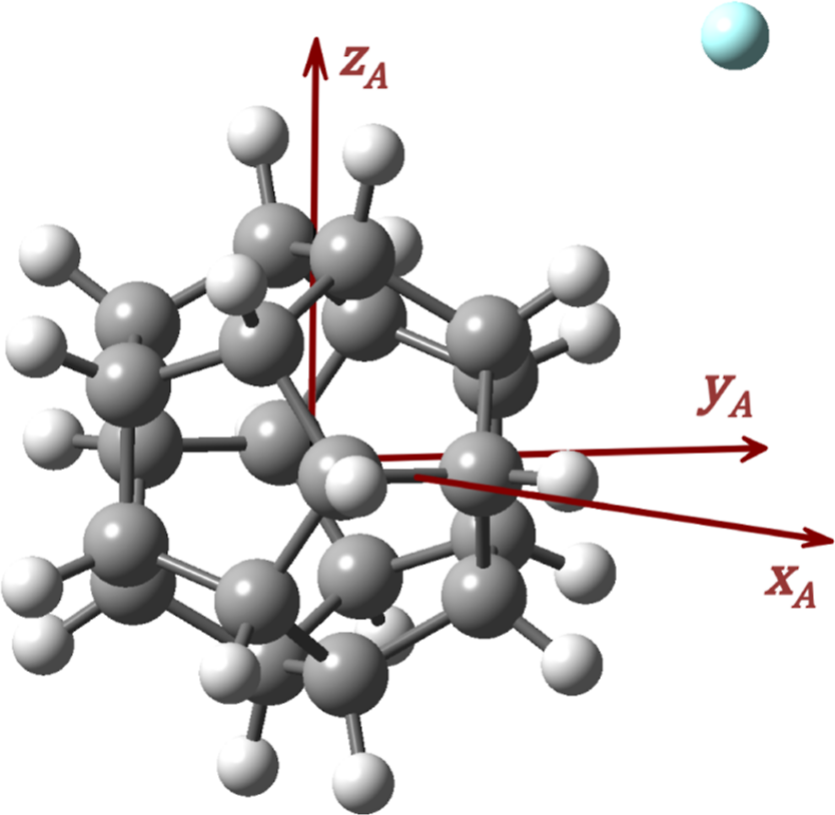
Dodecahedrane is shown interacting with the Ne atom, with C_20_H_20_ oriented according to LRF’s convention for *I*_h_ symmetry (see Table 2).

**Figure 14:**
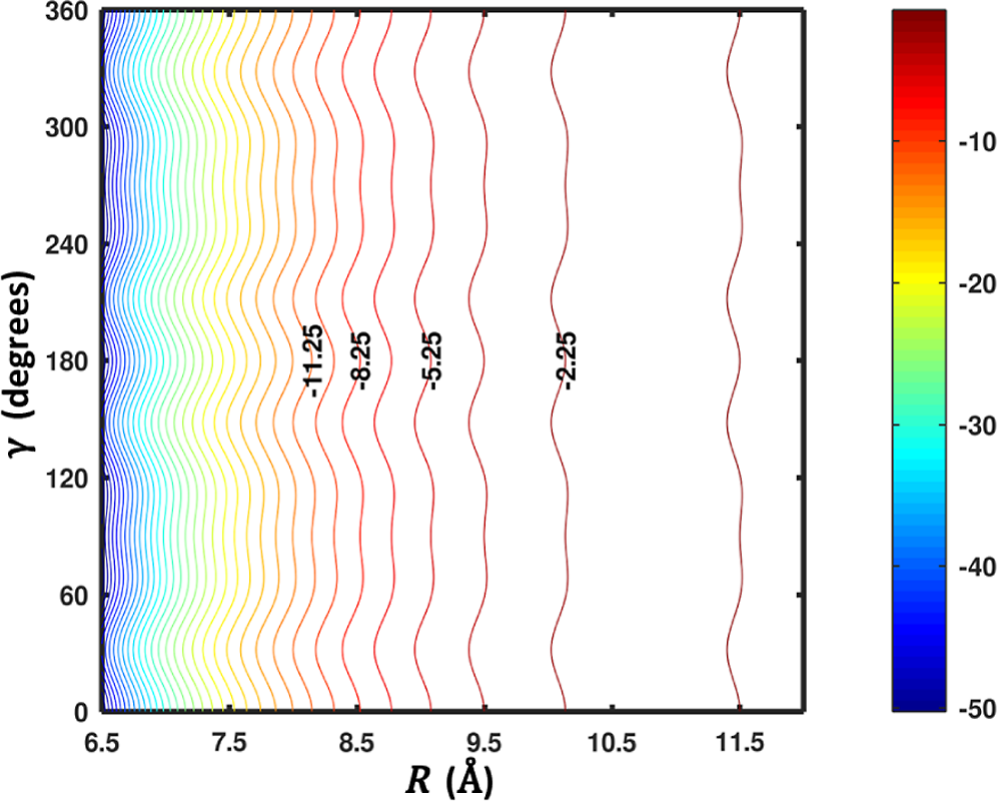
2D plot of R and γ with β fixed at 90 deg. Energy is plotted in units of cm^–1^. The modest, but significant anisotropy is apparent as well as the repeating pattern due to symmetry (see the text).

**Figure 15:**
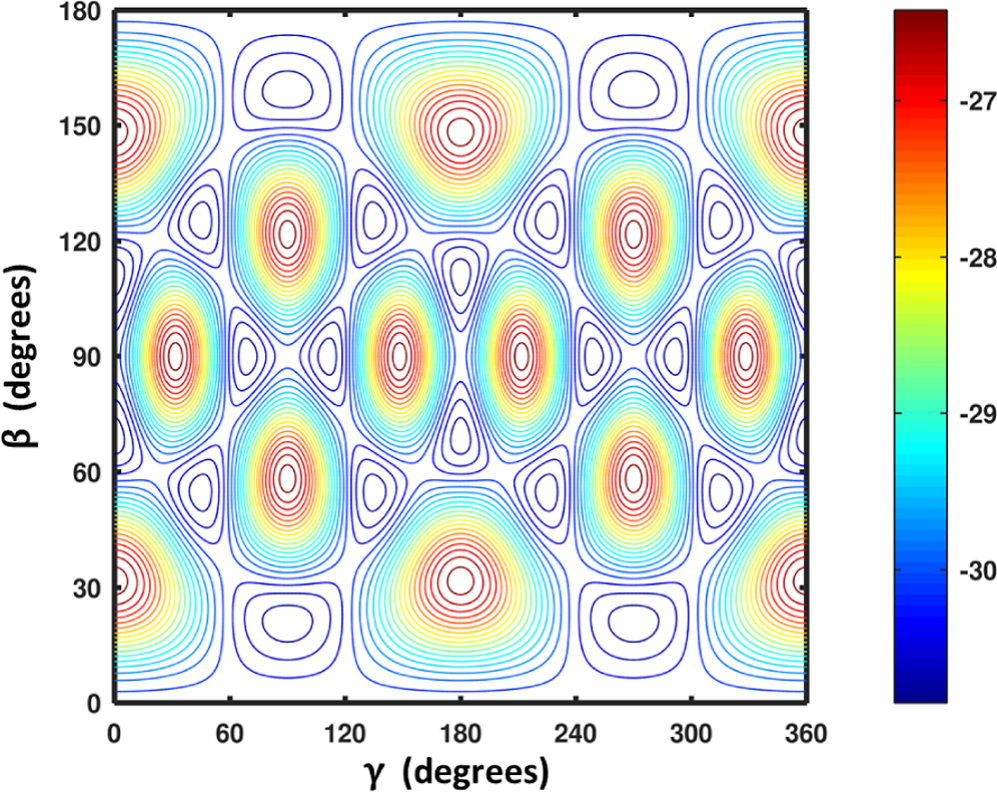
2D plot of γ and β, with R fixed at 7.0 Å. Energy is plotted in units of cm^–1^. The high symmetry and corrugated nature of the PES are illustrated (see the text).

## 4 Conclusions

We have introduced long-range-fit (LRF), a software package to construct and study a representation of LR molecular interactions based on a spherical tensor expansion of electrostatic, induction, and dispersion interactions. LRF can implement specified values of electrostatic moments and polarizabilities, or more typically, can process ab initio data from any electronic structure package and fit the coefficients for a selected set of interaction terms. The program can handle any two atoms or rigid molecules, charged or neutral, in ground or excited (nondegenerate) electronic states, and proper treatment is given to identical or chiral partners. LRF is adapted to all molecular symmetry point groups and upon specification of the two interacting species will produce a table of all relevant interaction terms, organized by electrostatics, induction, and dispersion, up to 15th-order (*R* ^–15^). Terms to be included in the expansion can then be specified by order for each interaction type.

LRF is built under a MATLAB GUI, which can be run on a WINDOWS PC and provides a user-friendly interface with various menus and check boxes to specify the workflow, with many options. For strongly anisotropic molecules, LRF includes an algorithm that goes beyond a simple radial cutoff when specifying the long range. The package also provides an analysis platform to obtain information and interpretation of the different interactions and their individual components. There are built-in tools to assess fit quality, provide outlier detection, PES visualization, and export efficient (FORTRAN) routines to allow evaluation of the LR interactions and integration with short-range PESs and/or dynamics packages.

We have highlighted some of the features and capabilities of LRF by treating four example systems that span cases of ions, extreme anisotropy, unusual electrostatics/dispersion, and high symmetry. LRF is designed as a convenient tool to extend any developed ab initio PES with an accurate long range, including as an easy update to existing PESs. Accuracy of the LR is especially relevant to spectroscopic and scattering studies in low-density and low-temperature environments such as the atmosphere, ISM, circumstellar environments, and atmospheres of moons, planets, and comets.

## Supplementary Material



## Notes

LRF user manual describing how to obtain and submit the signed license agreement and then the software, which is provided with two example data sets; installed file directory (S1); an image of the LRF dashboard following the two examples (S2 and S3); and an image illustrating how the exported fit can be called as a Fortran routine (S4) (PDF)
