# Supplementary material for: Long-Range Fit: A Software Package for the Representation and Study of Long-Range Molecular Interactions
Source: J Chem Theory Comput. 2026 Jan 27;22(3):1363–80. doi: 10.1021/acs.jctc.5c01984 (PMC12895421; doi:10.1021/acs.jctc.5c01984)
Supplement: Supplementary file 1 [file ct5c01984_si_001.pdf]

"Long-Range Fit: A software package for the representation and study of long-range molecular interactions"

User Guide

Adrian L. Batista-Planas,* Ernesto Quintas-Sánchez,* and Richard Dawes*

Department of Chemistry, Missouri University of Science and Technology,

Rolla 65409 MO, USA

* *Email: albgzz@mst.edu; quintassancheze@mst.edu; dawesr@mst.edu*

version 4.9

LRF: A Quick-Start Guide

Long-Range-Fit (LRF) is a MATLAB-based tool designed to facilitate the construction and study of the the long-range (LR) region of potential energy surfaces (PESs). In most foreseeable applications, LRF will be used to complement theoretical studies of molecular systems composed of two rigid molecules, for which a dataset of *ab initio* energies is being produced and extends into the LR region of the PES.

LRF has a MATLAB-based Graphic User Interface (GUI) freely available for non commercial purposes. Although based on MATLAB, the use of LRF does not require the installation of MATLAB nor a MATLAB license. Instead, a freely available MATLAB Runtime[*] library for Windows (version 2024a) is all that is needed to provide the full functionality of the app. This introductory guide describes how to obtain, install, and use LRF. This is a preliminary version of the User's Guide and some sections will be expanded in the future. Additional information can be found in the documentation released with the program.

[*]MATLAB Runtime is a collection of shared libraries, MATLAB code, and other relevant files needed to run compiled MATLAB applications on a target system without a licensed copy of MATLAB. All relevant documentation can be found in MathWorks website.

Contact Information

Team Members	Email
Richard Dawes	dawesr@mst.edu
Ernesto Quintas-Sánchez	quintassancheze@mst.edu
Adrian Batista-Planas	albplanas0904@gmail.com

Department of Chemistry, MS&T, Rolla, USA.

Prerequisites

Most applications of LRF are not very resource-intensive and can be performed on any modern PC, including laptops, provided the machine has at least a x86–64 processor, 4 GB of RAM, and the MATLAB Runtime library. If not already installed, the compatible version of the library will be automatically downloaded and installed by the provided LRF-installer ($\sim$ 3 Gb of available disk space and an internet connection are needed during the installation of the library); alternatively, the user can download and install the corresponding MATLAB Runtime version directly from the MathWorks website.

How to Obtain LRF

For noncommercial purposes, LRF is freely available to academic users—research institutes, universities, individuals—upon signing a License Agreement form. The form can be requested by email directly to the authors. Upon receiving the completed and signed document, a link to download the latest version of LRF will be provided. The following section contain instructions detailing the installation process.

How to Install LRF

The installation process is straightforward. After returning the signed license agreement, the user will receive an email with a link to download a compressed ZIP file; which, upon extraction, should create a new directory called LRF, which structure is shown in Figure S1.

```
LRF/
├ INSTALL/
│  └ LRF-install.exe
├ DOC/
│  ├ AUTHORS.txt
│  ├ CHANGES.txt
│  ├ COPYRIGHT.txt
│  ├ INSTALL.txt
│  └ start-guide.pdf
├ EXAMPLES/
│  ├ CFp-H2.zip
│  └ C10Hm-H2.zip
├ SOURCE/
│  └ Fortran codes
└ README.txt
```

Figure S1: LRF directory tree.

To install:

1. **Execute the Installer**: Double-click on the installer file ("`LRF-install.exe`", located in the folder "`LRF/INSTALL/`") to start the installation process. Administrator privileges are required to execute the MATLAB Runtime installer. During installation, warning dialogues may appear due to Windows' system-settings and antivirus software, as the application is not listed in the Microsoft Store.

2. **Choose Installation Location**: Use the default path, or click "Browse" to select a different folder.

3. **Accept License Agreement & Install**: Carefully read the terms and conditions, and click "Next". The MATLAB License Agreement must be accepted to continue. The installation could take several minutes.

How to Use LRF

The nonlinear fitting algorithms employed by LRF scales in cost with both the size of the data set and, more importantly, the number of fitted terms—which increases rapidly with the order in the multipole expansion; thus, running times (to fit the data) may range from a few seconds to several minutes depending on the system's complexity and the available computing resources. As illustrated in the examples, it is recommended to begin with a small number of leading terms and then extend sequentially by order, updating the fit at each stage. This iterative approach allows to monitor the quality of the fit through error residuals and related diagnostics available in the app, thereby informing the user of the optimal point at which to terminate the fitting process with satisfactory results.

Testing the LRF Installation

After the installation is complete, launch the LRF app using the Desktop icon. It is suggested to explore the directory: "`LRF/EXAMPLES/`", where all the necessary input files and generated output files are provided for two example systems. The easiest way to check the installation, is to replicate these basic calculations—which will test the basic functionality of LRF.

CF^{+}–H_2 system

In this example, we consider the interactions between positively charged CF^{+} ($C_{\infty \mathrm{v}}$ symmetry) and neutral H_2 ($D_{\infty \mathrm{h}}$ symmetry). All relevant information (*ab initio* data file and final set of fitted coefficients) is included in the file '`CFp-H2.zip`' (located in the folder '`/LRF/EXAMPLES/`').

Step-by-step procedure:

1- System Setup & Initialization: Define the system and load *ab initio* data.

- *Working Directory.* Choose or create a local folder for logs and outputs.
- *System Definitions.* Name: "`CFp-H2`"; symmetry of fragment A: "$C_{\infty \mathrm{v}}$"; charge of fragment A: "1"; symmetry of fragment B: "$D_{\infty \mathrm{h}}$"; charge of fragment B: "0".

- *Input data file.* Select file `CFp-H2-Abinitio.dat` (included in the provided ZIP file), the dataset is in a six-column format:

$$\text{index} \qquad R\ [\text{Å}] \qquad \cos\beta_1 \qquad \cos\beta_2 \qquad \alpha\ [\text{rad}] \qquad E\ [\text{kcal/mol}]$$

  Select the corresponding columns and units as above (as shown in Figure 4 of the paper). This format is denoted AUTOSURF, but user constructed data files with the same column structure can be used.

- *Initialization.* Finally, press "Initialization" (red button). LRF will generate and store the complete case-specific expansion up to 15th order. This only takes a few seconds. Tables of all relevant terms in the expansion are then produced, and can be seen in the 'EXPANSION' tab, as shown in Figure 5 of the paper. LRF will also process the dataset to estimate initial values for the first few coefficients in the fitting. Once the initialization is complete, LRF will move automatically to the 'FITTING' tab, Figure S2.

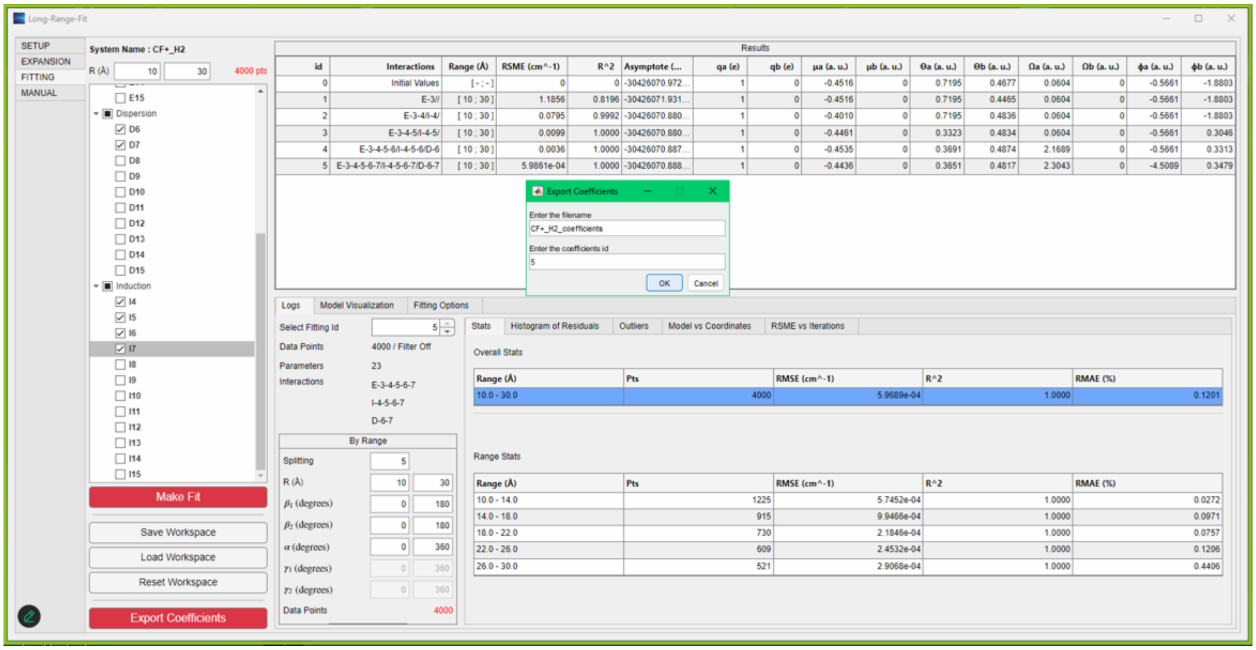

Figure S2: 'FITTING' tab. After completing the fitting process, export the coefficients.

2- Fitting the expansion: For each order n, all terms from **Electrostatic (E)**, **Induction (I)**, and **Dispersion (D)**, can be included using the checkbox menu in the left, then

press the button 'Make Fit'. In our experience, the most robust way to proceed towards a satisfactory fit is to introduce the terms of the expansion order by order, updating the fit at each step and monitoring the behavior and statistics of the fit, until sufficient accuracy is achieved. Each fit is stored in a new row of the Results table (as can be seen in Figure S2). The user can test up to higher-order fits, but chose to export and use any lower-order fit instead. We suggest increase the order until the fit performance plateaus, and retain/export the prior order.

For this particular test scenario, with the provided data set, the following procedure:

1. include Order 3, $\mathbf{E_3}$: $q^A\,\Theta^B$ (leading electrostatic).
2. include Order 4, $\mathbf{E_4}$: $\mu^A\,\Theta^B$. $\mathbf{I_4}$: $(q^A)^2\,\alpha^B_{\mu\mu}$ (leading induction).
3. include Order 5, $\mathbf{E_5}$: $q^A\,\Phi^B + \Theta^A\Theta^B$. $\mathbf{I_5}$: $q^A\,\mu^A\,\alpha^B_{\mu\mu}$.
4. include Order 6, $\mathbf{E_6}$: $\mu^A\,\Phi^B + \Omega^A\,\Theta^B$. $\mathbf{I_6}$: $q^A\,\Theta^A\,\alpha^B_{\mu\mu} + (\mu^A)^2\,\alpha^B_{\mu\mu} + (q^A)^2\,\alpha^B_{\Theta\Theta}$.
   $\mathbf{D_6}$: $D_{\mu\mu-\mu\mu}$ (leading dispersion).
5. include Order 7, $\mathbf{E_7}$: $q^A\,\xi^{(6)B} + \Theta^A\,\Phi^B + \Phi^A\,\Theta^B$. $\mathbf{I_7}$: $q^A\,\Omega^A\,\alpha^B_{\mu\mu} + \mu^A\,\Theta^A\,\alpha^B_{\mu\mu} + q^A\,\mu^A\,\alpha^B_{\Theta\Theta}$.
   $\mathbf{D_7}$: $D_{\mu\Theta-\mu\mu}$.

should provide results similar to those shown in Figure S2. Notice that numerical differences in the output are normal, due mainly to the nature of the non-linear fit performed to obtain the coefficients. However, if the results are significantly different, first check the 'SETUP' tab and confirm that the fragment symmetries and charges were entered correctly, and that the correct units are specified for the energies in the data file. Also, on the 'FITTING' tab, confirm that the *min* and *max* R values correspond to the desired range—if a datafile for the entire PES is loaded, then a reasonable value for the min R is needed to exclude the close interaction region, where the LR multipole expansion isn't valid. For this particular example, the min value of R is 10 Å.

3- Export long-range coefficients

- Press "Export Coefficients" button.
- Enter a file name

- Press “Save”.

$C_{10}H^{-}$–H_2 system

In this example, we consider the interactions between negatively charged $C_{10}H^{-}$ ($C_{\infty v}$ symmetry) and neutral H_2 ($D_{\infty h}$ symmetry). All relevant information (*ab initio* data file and final set of fitted coefficients) is included in the file ‘`C10Hm-H2.zip`’ (located in the folder “`/LRF/EXAMPLES/`”). The step-by-step procedure is the same as in the previous example, except for the charge of fragment A (which should be set to “−1”) and the fitting procedure, which is carried out up to 10th order in this case, as shown in Figure S3.

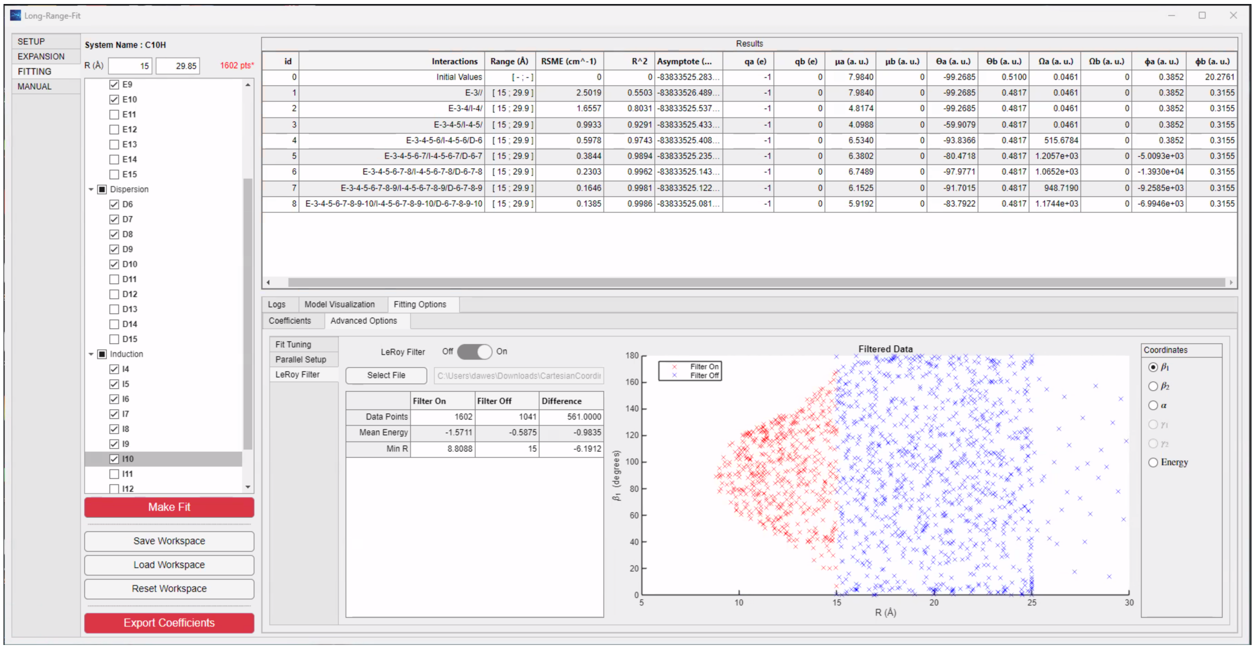

Figure S3: After completing the fitting process, export the coefficients.

For this system, a “LeRoy Filter” option is implemented as discussed in the paper. As shown in Figure S4 (upper panel), this is specified in the “Advanced Options” of the “Fitting Options” tab, and includes uploading a Cartesian coordinate file (the file for this case is included in the ZIP) for the fragments (“Select File” button). This allows the data points indicated in red (in the right side of the image) to be included in the fit, rather than defining the onset of the long range at a fixed value of R. The minimum value for R, specified in the top left of the ‘FITTING’ tab (cf. Figure S3), above the fitting terms, should be chosen as the minimum R for which all points are to be used. The algorithm will then determine the additional points (for lower values of R, highlighted in red) to be included in the fit. In

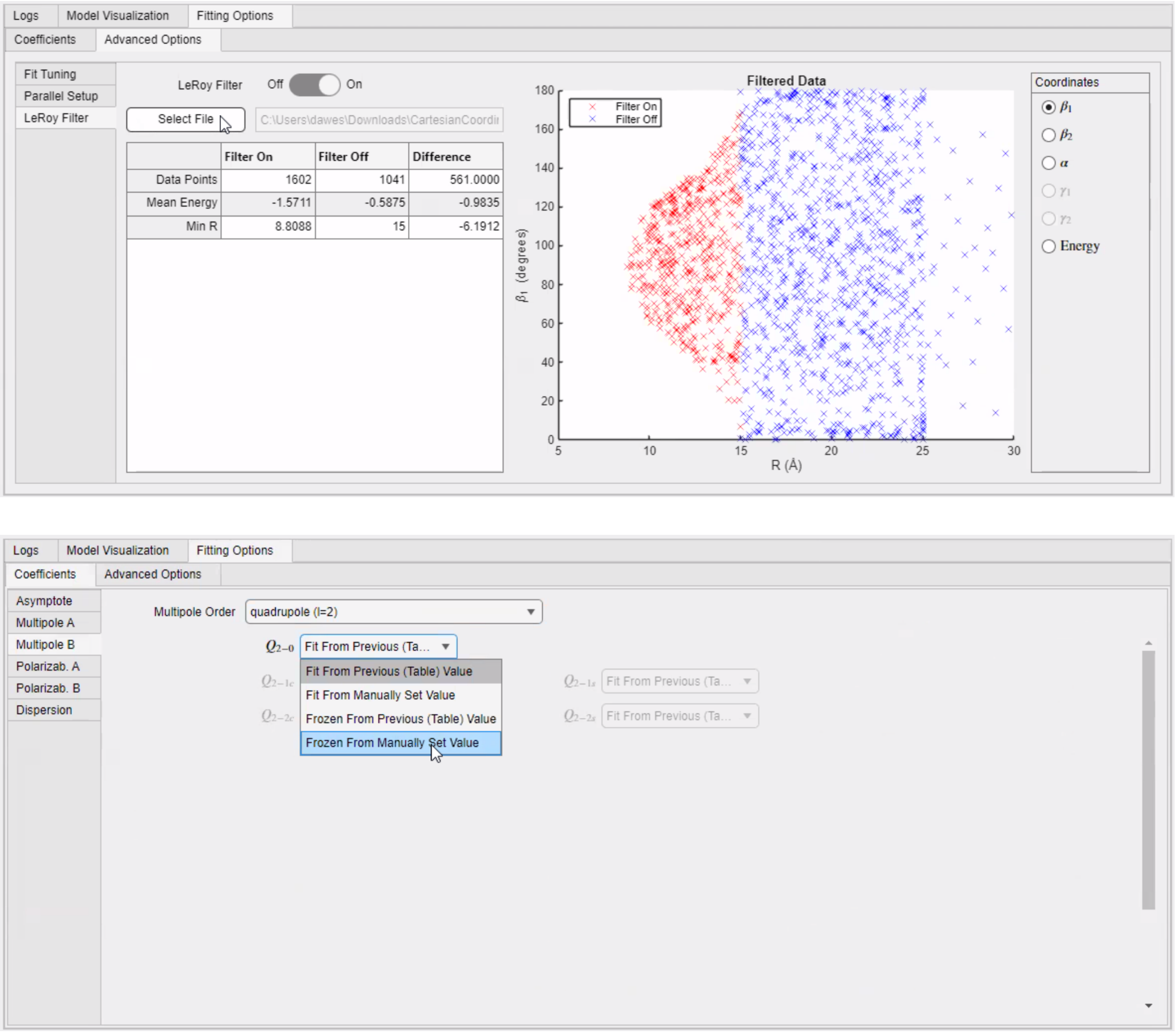

Figure S4: Implementing the LeRoy Filter (upper panel) and/or fixing coefficients to known values (lower panel) is demonstrated.

this example, the capability to input known values as fixed coefficients is also demonstrated. As shown in the lower panel of Figure S4, quantities can be set as “Frozen From Manually Set value”. For this system we set the quadrupole and hexadecapole of H_2 to the values of 0.4817 and 0.3155 respectively (as obtained by fitting the CF^+ system in the previous example. Since these are sensible values, they don’t impair the fit, but they do speed up the fitting slightly since there are fewer free parameters.

Evaluate the fitted LR-PES

Once the coefficients are saved, copy the FORTRAN codes "`LRF.f90`" and "`min-example.f90`" (included in the directory "`LRF/SOURCE`") into the folder containing the coefficients-file. The file "`LRF.f90`" contains all the necessary subroutines, and the program "`min-example.f90`", shown below, provides a minimalistic example of how to evaluate the fitted PES for a single configuration. Notice that the variable "`COEFFICIENTS_FILE`" should be modified to match the name of the coefficient's output file produced by LRF. The variable "'`XDIM`" (set to 4 in this example) should also be modified if needed to match the dimensionality of the system. To test this minimal example, open the folder in a terminal, compile the source code and create the binary:

```
gfortran LRF.f90 min-example.f90 -o min-example
```

Upon execution, the value of the potential corresponding to the selected geometry (specified by the vector "`coordinates`") will be printed.

```fortran
PROGRAM LRF_min_example
 implicit none
 integer (kind=4), parameter:: XDIM=4 ! Dimensionality of the system
 character (len=*,kind=1):: COEFFICIENTS_FILE
 real (kind=8):: energy, coordinates(XDIM)

 ! coefficients output-file produced by LRF
 COEFFICIENTS_File = "./coefficients-file.txt"

 ! same coordinates as in LRF ab initio data file
 ! (but always Ang. for distances and deg. for angles)
 coordinates(1) = 10.0d0  !R (in Angstroms)
 coordinates(2) = 30.0d0  !beta1 (in degrees)
 coordinates(3) = 20.0d0  !beta2 (in degrees)
 coordinates(4) = 120.0d0 !alpha (in degrees)

 ! Evaluate the fitted PES
 call evaluate_LRF(energy,XDIM,coordinates,COEFFICIENTS_FILE)

 write(*,*) "value of the potential: ", energy

END PROGRAM LRF_min_example
```
